# The retention benefits of cumulative versus non-cumulative midterms in introductory biology may depend on students’ reasoning skills

**DOI:** 10.1371/journal.pone.0250143

**Published:** 2021-04-22

**Authors:** Elizabeth G. Bailey, Rebeka F. Greenall, Madeleine M. Tullis, Kurt R. Williams

**Affiliations:** Department of Biology, Brigham Young University, Provo, Utah, United States of America; Universidad de Chile, CHILE

## Abstract

Assessment has long played an important role as a measurement tool of student mastery over course content. However, testing has also been shown to be an effective learning tool. Cumulative testing, in which all material from the entire learning period is covered, has been assumed to be effective, yet few studies have explicitly tested its effectiveness compared to non-cumulative testing. Studies in psychology and mathematics courses suggest that cumulative final exams increase long-term retention of information, and cumulative testing during the semester can increase cumulative final exam performance and long-term retention. Because frequent testing has also been shown to increase student learning, the purpose of this quasi-experimental study is to investigate the effects of cumulative versus non-cumulative midterms on student learning in a course that uses frequent assessment. In this study, one section of an introductory biology course for non-majors was given seven cumulative midterms, with about half of the questions drawn from previous units and the rest covering the current unit. The other section was given seven non-cumulative midterms that focused on current material while other course characteristics were held constant. Student performance on a common, cumulative final exam and a retention exam five months later were compared. Midterm format had no effect on final exam performance, contradicting the few studies done in psychology and mathematics courses. Thus, there may be no additional benefit of cumulative testing if exams are given frequently. Cumulative midterms appeared to increase retention after five months, but only for students who entered the course with low reasoning skills. Interestingly, students with high reasoning skills appeared to retain more from the course if they were given non-cumulative midterms. Possible explanations and ideas for future research are discussed.

## Introduction

The idea that assessments drive learning, and should thus be carefully designed, is a widespread idea in higher education, yet there is still so much about the effects of various kinds of assessment that we do not understand [1]. Surprisingly, few studies have explicitly tested the benefits of cumulative exams, defined as tests that include material taught during the entire learning period–often a term or semester. Based on the ideas of the testing effect and spaced study, we would expect that cumulative exams would increase student learning and retention more than non-cumulative exams that only cover the most recent unit of the course. However, the benefits of such exams may vary based on other course and student characteristics.

### Previous research on cumulative testing

Two studies suggest that cumulative final exams increase longer-term retention. University psychology students retained more four to five months after the final exam on question items that had been tested on a cumulative final exam compared to questions that were not tested on the final exam [2]. Another study done in university psychology courses found that students who took cumulative finals retained more knowledge compared to students who took non-cumulative finals, and this was true both directly after the final exam and up to 18 months after the course [3].

Evidence also suggests that cumulative exams during a semester can increase performance on a cumulative final exam. One study found that university introductory mathematics students who were given cumulative tests throughout the semester performed better on a cumulative final exam at the end of the semester compared to a control group with non-cumulative exams [4]. Another study done in an introductory psychology course investigated the impacts of cumulative testing throughout the semester on both the final exam and a retention exam two months after the course ended. Similar to the study described above, students who took cumulative exams throughout the semester performed higher on the final exam. However, longer-term retention effects two months later differed by student population: high-scoring students’ retention was unaffected by the type of exams they took throughout the semester, but low-scoring students remembered more of the course material if they took cumulative tests [5]. The benefits of cumulative testing might also vary by population. Cumulative testing has been suggested to be more beneficial for students who have high self-directedness compared to those with low self-directedness [6], introductory students compared to upper-division students [3], and perhaps lower-performing students compared to high performers [4, 5, 7].

We previously tested the benefits of cumulative testing in an introductory biology course and found that student performance on high-level test questions requiring data analysis increased when students took 10 short, cumulative midterm exams compared to the original two non-cumulative midterms [8]. However, with that study design, it was impossible to tell whether students benefitted most from the high frequency of testing, the cumulative nature of those tests, or both. A similar issue applies to other studies conducted in a microeconomics undergraduate course and in a medical school. Students with frequent cumulative testing throughout the semester outperformed students who were only given a cumulative final exam, but it is unclear whether the benefit arose from more frequent testing, cumulative testing, or both [9, 10]. A meta-analysis of classroom-based studies in math, science and the social sciences showed that student performance increased with the number of tests given and that students who took a large number of short tests benefited more than students who took a small number of long tests of identical items [11]. Thus, separating out the effects of frequent assessment and cumulative assessment is needed.

### Theoretical foundation for cumulative testing

#### Testing effect

The testing effect, whereby testing and retrieval increases learning and retention better than repeated studying, has been investigated since the early 1900’s [12, 13]. The theoretical rationale behind test-enhanced learning includes both direct effects of retrieval, described here, and indirect benefits of testing, described below under *Test Expectancy*. The retrieval process itself can be responsible for increased learning [14] as different routes of access are created during the testing experience and encoding becomes deeper or more effortful [15, 16]. Retrieval effort theories thus predict larger testing effects for recall tests than recognition tests, such as multiple-choice, due to the greater effort required for free recall, and data from various laboratory studies support this idea [16]. Different modes of testing have also been studied in the classroom, and short answer tests have been shown to benefit student learning more than multiple-choice tests, although multiple-choice tests were still beneficial [17]. There are potential negative impacts of frequent testing when questions are in a multiple-choice format due to the incorrect lures, but studies suggest that the positive benefits of tests, even with multiple-choice questions, outweigh potential negatives [18]. Furthermore, results from university students also show that properly constructed multiple choice questions with plausible alternatives still require retrieval of memory regarding both the correct and the incorrect options and can thus produce a large testing effect [19].

Many laboratory studies have demonstrated the positive effects that retrieval opportunities have on learning and retention for low-level tasks like memorization [20–26] as well as transfer of knowledge to novel questions and higher-order thinking [27–31]. Although more recent, the testing effect has also been shown to be relevant to classroom environments and more authentic educational materials. Middle schoolers performed better on exams after being quizzed on material compared to being presented with the data twice [32], and quizzing increased performance on exam questions compared to a no-quiz control even when the test required a novel application of the content [33]. University students taking extra quizzes on textbook material memorized facts better than those who just read in a brain and behavior course [17], and anatomy and physiology students learned more by self-testing than re-reading and note-taking [34]. Balch found that introductory psychology students performed better on a final exam if they actually took a practice exam rather than studying the key of the same questions [35].

Evidence for the testing effect has also been found in the university biology classroom with higher-order skills. We previously found that average student success only plateaued after six retrieval opportunities for exam question requiring analysis of data [8]. In an introductory biology course, students who took high-level midterms performed better on high-level final exam questions compared to those who were only tested with low-level questions during the semester [36]. A follow-up study on open book exams verified that this testing effect on high-level questions involved the actual cognitive processes, not just practice on the low-level content [37].

#### Test expectancy

Testing can also increase learning indirectly as students prepare for a test [38] or respond to feedback after a test and adjust their studying before another exam [15]. Test expectancy describes the phenomenon whereby learners adapt encoding strategies and monitor their own learning based on the type of test they are expecting to take. In the laboratory, subjects who were expecting a final recall test benefited from that test more than those who were not expecting it, suggesting that students may continue mental processing of material so that it can be more accessible for an exam [39]. Also in the laboratory, learners who knew what format of test they would be taking outperformed those who did not [40, 41]. The idea of test expectancy altering student study habits has also been considered in an undergraduate biology classroom in regard to the cognitive task level students are expecting. Students who were given exams requiring higher-order cognitive skills may have studied differently than those who knew they would only be required to demonstrate memorization and understanding [36].

If students expect cumulative exams rather than expecting to only be tested on the most recent unit, they would be expected to allocate their study time differently in terms of course topics. A pilot study in a macroeconomics course supports this idea, as students self-reported that they altered their study behavior in courses that had cumulative final exams and spent more time reviewing previous material than they would have if a non-cumulative final were given [42].

#### Spaced study

The spacing effect, whereby long-term retention is increased by distributing study over multiple sessions rather than massing learning into a single session, is a well-documented phenomenon in both the laboratory and classroom (reviewed in [43]). In their meta-analysis, Cepeda et al. found that learning should be distributed across weeks or months if retention longer than one month is desired and that the optimal length of time between spaced sessions likely increases with the desired retention period [44]. Susser and McCabe [45] found that students generally believe that spaced studying will work, but students still report only intermediate levels of spaced studying. They were more likely to space their study if material was difficult, valuable, interesting, or worth a lot of points. Distributed testing and study can be used jointly, as testing students will likely motivate them to study. Cull discusses the difficulty of untangling repeated testing and spaced study, but found that university psychology students benefited most from both distributed studying and testing in combination [46].

### Our hypothesis

We used a quasi-experimental design to test the benefits of cumulative midterm exams compared to non-cumulative midterms in an introductory biology course that utilized more frequent testing. Two sections of the course were treated identically as much as possible, but one section took seven cumulative midterms while the other took seven non-cumulative midterms focused on the current unit. We hypothesized that frequent cumulative midterms increase both short- and long-term retention compared to non-cumulative exams. If this hypothesis is true, we would predict that students who took cumulative midterms would earn higher scores on both a cumulative final exam and on a cumulative retention exam five months after the end of the course. If cumulative midterms increase retention due to the testing effect, then we would predict that the benefit would be greater for earlier topics and learning objectives that were tested multiple times. If cumulative midterms increase retention due to differential test expectancy, then we would expect the benefits would be greater for students who repeatedly space their study throughout the semester in preparation for each midterm rather than waiting to re-study until right before the final exam.

## Methods

### Ethics statement

Written consent was obtained from all participants, and permission for use of human subjects was obtained from the Brigham Young University Institutional Review Board.

### Course description

Biology 100 is an introductory biology course offered at a large, 4-year private university for non-majors as part of the university’s general education requirement. The course was designed to introduce students to disciplines in the life sciences and give them basic literacy in the language of science and biology. The course covered biology concepts from biochemistry to ecology. The class met three times a week, and the instructor (author EGB) strove for a student-centered classroom using instructional methods that included interactive lecture with frequent formative assessment, clicker questions, and think-pair-share. Students were given seven short midterm exams throughout the semester, and these scores may or may not have counted towards their final grade according to a unique grading scheme designed to promote a growth mindset (described in [8]). The midterm exams were taken in class, and students received feedback immediately after the exam as they went over the answers together.

### Study design

**Fig 1:**
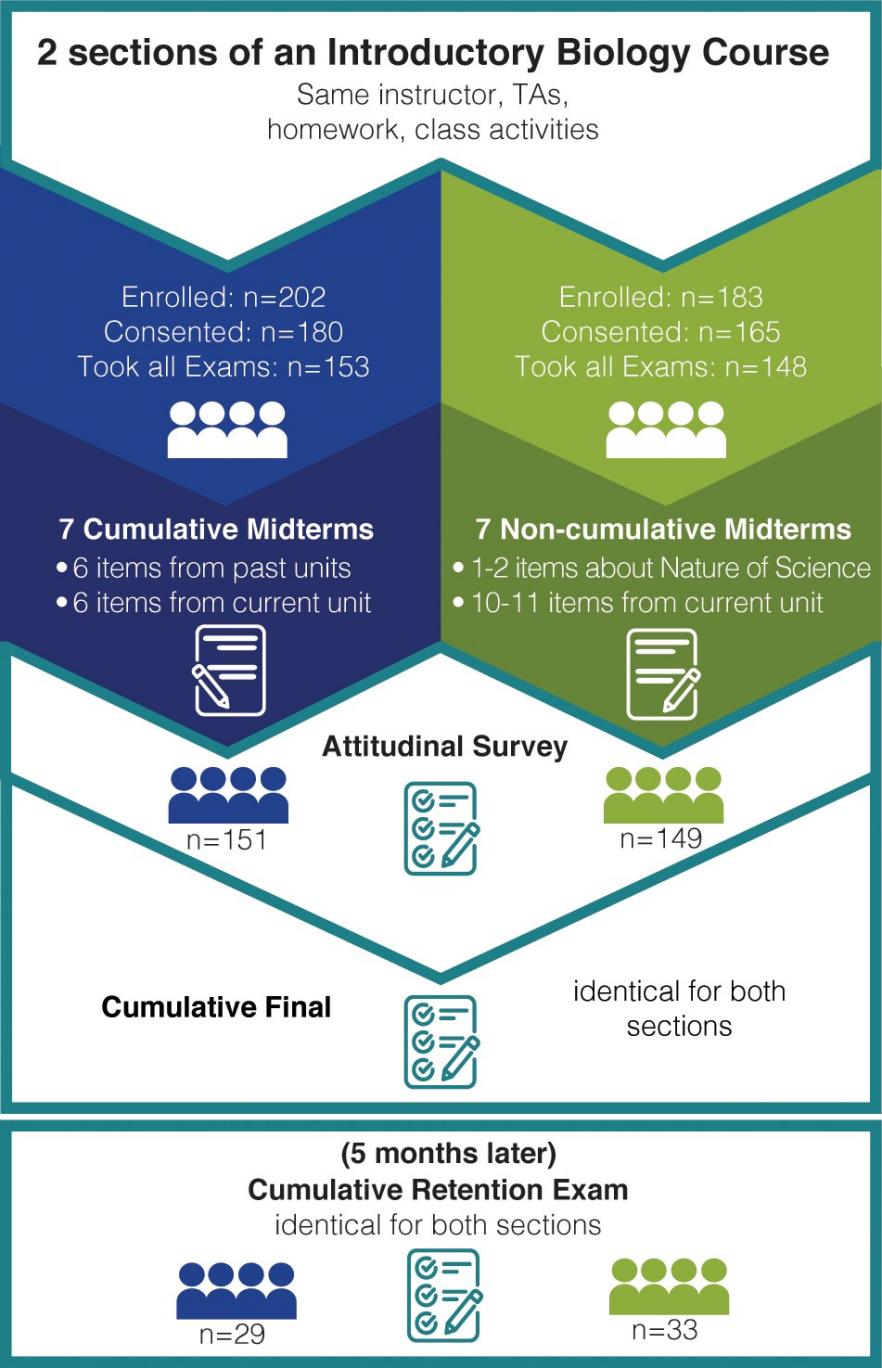
Quasi-experimental study design. Blue represents the cumulative section, and green represents the non-cumulative section.

We implemented a quasi-experimental study design (shown visually in Fig 1) using two sections of the course during Fall semester of 2015. To consider section equivalence, we used the content-independent Lawson Classroom Test of Scientific Reasoning (LCTSR; version from 2000, with 24 items, including four items aimed at postformal reasoning; [47, 48]) to assess students’ scientific reasoning ability at the beginning of the semester. All instructional techniques were identical between sections (same lectures, same assignments, same class activities, same instructor, same teaching assistants, etc.), except one section was given cumulative midterm exams while the other took non-cumulative midterms. Each midterm contained 12 multiple-choice items: cumulative midterms had about six questions drawn from previous units and the other six questions covering the current unit, while non-cumulative midterms included one or two items about the nature of science (e.g., drawing conclusions from experimental data), and 10–11 items from the current unit. We recognize that the items about the nature of science make the non-cumulative midterms somewhat cumulative, but the instructor still chose to include those items as the nature of science was meant to be a constant thread throughout the course. Although the midterm exams were different, we included as many identical items as possible between the two sections. The first midterm was completely identical, and the subsequent midterms contained 7–8 identical questions for the two sections (6 on the current unit and one or two on the nature of science). When cumulative midterms covered previously tested material, the test questions covered the same learning objective but were not identical to previous questions (see S1 Appendix for examples).

At the end of the semester, both sections completed the same survey about their attitudes toward the midterm exams and their study habits. Both sections then completed the same cumulative final exam consisting of 48 multiple-choice items (9 remember or understand questions; 12 low-level apply questions; and 27 high-level apply, analyze, or evaluate questions; [49, 50]). All course topics were covered using roughly the same number of test items, and all test questions were novel (i.e., students had never seen the questions on previous exams).

Students from both sections of the course were invited by email to return and take a retention exam about five months after the final exam. This time period was chosen because we wanted a full semester to have passed, but we did not want to wait so long we could not get students to return. Participants were offered a small amount of compensation and told that this exam would not impact their original course grade. They were also instructed not to study. Overall, 16% of consented students returned from the cumulative section and 18% returned from the non-cumulative section. While all students were invited, the students who elected to return did not necessarily reflect the characteristics of the larger student sample. Students who returned to take the long-term retention exam (*M* = 20.66, *SD* = 2.74) had significantly higher LCTSR scores, *t*(121.14) = 5.76, *p* < 0.0005, compared to students who did not return (*M* = 18.22, *SD* = 4.17) when compared by unequal variances t-test.

The retention exam was made up of 24 items that were very similar in nature to 24 times on the cumulative final exam but not identical (two remember/understand questions; five low-level apply questions; and 17 high-level apply, analyze, or evaluate questions; [49, 50]). We also included a short attitudinal survey at the end of the exam to see if students remembered which midterm format they had been given during the semester and which treatment they thought would lead to greater retention.

#### Participant inclusion and sample sizes

As shown in Fig 1, the section that took cumulative assessments had 202 students enrolled, but only 180 students gave consent for their data to be included in our study. Our dataset is not complete for all of the students who gave consent. For example, only 177 of those students took the LCTSR, 153 took every midterm and the final exam, and 151 took the attitudinal survey at the end of the semester. Finally, 29 students agreed to return five months after the final exam to take a retention exam. The section that took non-cumulative assessments had 183 students enrolled, and 165 of those students gave consent for their data to be included in our study. However, only 162 students took the LCTSR, 148 students took every midterm and the final exam, and 149 students took the attitudinal survey at the end of the semester. After five months, 33 students agreed to return and take the retention exam. We included as many students in each analysis as possible, but some analyses have lower sample sizes due to incomplete data. For this reason, sample sizes are listed for each analysis.

#### Exam question classification

All test questions were classified by two raters independently based on the revised Bloom’s Taxonomy [49, 51]. We separated “apply” questions into two categories in order to classify each exam question as requiring lower-order cognitive skills (LOCS) or higher-order cognitive skills (HOCS) as described in the Blooming Biology Tool [50, 52]. Two raters classified questions individually, achieving 86% agreement (Cohen’s kappa = 0.71) on LOCS/HOCS classification and 88% (Cohen’s kappa = 0.81) on Bloom’s classification. After independent classification, the two raters discussed any questions on which they initially disagreed until they came to agreement. Occasionally a third rater was brought in to help resolve disagreements, especially those surrounding apply questions and whether they required LOCS or HOCS. Finally, all three raters agreed that an apply question would be rated as a HOCS question if it required two or more cognitive steps to solve and a LOCS question if only one cognitive step was necessary (or if there were two steps taken but the second step could be avoided or replaced with a memorized algorithm). We maintained that searching for relative information in a long word problem did not count as a cognitive step. Example test items and their classifications are found in the S2 Appendix.

#### Study habits

In the end-of-course attitudinal survey, we asked students how many hours they studied per week on average, how many midterm exams they studied in preparation for the next midterm exam (none, some, all), and how many midterm exams they studied in preparation for the final exam (none, some, all). As a measure of the quality of their midterm exams review, we asked them to pick all that applied from the following options (listed here in order of quality): “I noted which problems I missed and what topics I needed to review,” “I tried to understand WHY the correct answer was correct,” “I tried to understand WHY the wrong answers were incorrect,” and “After reviewing the question, I tried another practice problem of the same type.” We converted that order of quality on a scale from 1–4 (with noting which problems you missed as the lowest quality = 1 and trying another practice question of the same type as the highest quality = 4). Since students could mark more than one option, the highest quality option they marked was recorded as their review quality score.

### Statistics

When we performed linear mixed models, we used Akaike’s Information Criterion corrected for small sample sizes (AICc) for model selection. Models within an Information Criterion of 2 were considered equivalent, and if they were within 2, the model with the fewest number of parameters was chosen as the best model. All analyses were performed using the linear mixed models function in IBM SPSS Statistics (version 27). In brief, we generally used the method described by Theobald [53] for all linear mixed models:

1. First, all possible fixed effects were included, and restricted maximum likelihood estimation was used to select random effects. The random effects included in the best-fitting model were retained for the rest of the analysis. (We considered including random intercepts for *student* and *topic*. In all cases, including a random intercept for both *student* and *topic* improved the model. Thus, both are included as random effects in all final models.)
2. Random effect inclusion was validated by calculating the intraclass correlation coefficient of each random effect in an empty model.
3. Fixed effects were then selected using maximum likelihood estimation.
4. Finally, the best model was refit using restricted maximum likelihood estimation to get the most precise parameter estimates.

For all mixed models, only statistics from step 4 are included in the main text but results of steps 1–3 are included in the Supporting information.

When interactions were included in multiple linear regression, variables were centered around their mean before interaction terms were calculated in order to avoid multicollinearity with their component variables. Whenever we used backward stepwise multiple linear regression, the variable with the largest probability of F was removed for each step as long as *p* > 0.1 (F test). When box plots are shown on graphs, boxes represent the median and quartiles while the whiskers show the full range of data. All other statistical methods are described in the text and/or figure legends.

## Results

### Equivalence of sections and midterms

We implemented our treatment in two sections of the same introductory biology course for non-majors, with one section taking cumulative midterms while the other took non-cumulative midterms. Due to the quasi-experimental nature of our study design, we first investigated the equivalence of our two sections. As shown in Table 1, the two sections were considered statistically equivalent before the course began in terms of scientific reasoning ability (LCTSR) at course entry, year in school, interest in biology before the course, major, and gender. Most of these student characteristics were still considered in regression analyses later in the study, but year in school and STEM major were not used in regression analyses because of the large amount of missing data for these two variables. Table 1 also shows general equivalence of sections in terms of non-assessment grades in the course: attendance, writing assignments, reading assignments, and other homework assignments. The difference between the two sections in terms of reading assignments was close to significant, with the cumulative section completing fewer of these assignments. That potential difference will be considered later on in regression analyses as part of the “preparation” variable described below.

**Table 1:** Sections were generally equivalent.

Variable	Cumulative			Non-Cumulative			Statistical Test	*p*
Major ^a^	65 STEM, 95 not			46 STEM, 96 not			Fisher’s exact	0.15
Gender ^a^	100 male, 80 female			86 male, 79 female			Fisher’s exact	0.59
	**Mean**	**SD**	**N**	**Mean**	**SD**	**N**		
Scientific Reasoning ^b^	18.76	4.09	177	18.69	3.88	162	Ind. samples t	0.88
Year in School ^a^	1.65	0.90	159	1.47	0.72	142	Mann Whitney U	0.16
Pre-Interest in Bio ^a^^c^	2.83	1.08	151	2.84	1.05	149	Mann Whitney U	1.00
Attendance ^d^	97.03	7.85	180	96.82	7.15	165	Ind. samples t	0.80
Writing Assignments ^d^	91.38	14.70	180	90.45	13.24	165	Ind. samples t	0.54
Reading Assignments ^d^	89.51	14.75	180	92.22	11.47	165	Welch’s t	0.06
Other Homework ^d^	88.25	17.78	180	89.37	15.93	165	Ind. samples t	0.54

^a^ Self-reported

^b^ Assessed at the beginning of the semester using Lawson’s classroom test of scientific reasoning

^c^ Data self-reported

^d^ Scores are reported as percentage points earned by the end of the semester

The two sections were also equivalent in the returning sample of students who took the retention exam (S1 Table), but high-scoring students and STEM majors from both sections were more likely to return for the retention exam (S2 Table).

**Fig 2:**
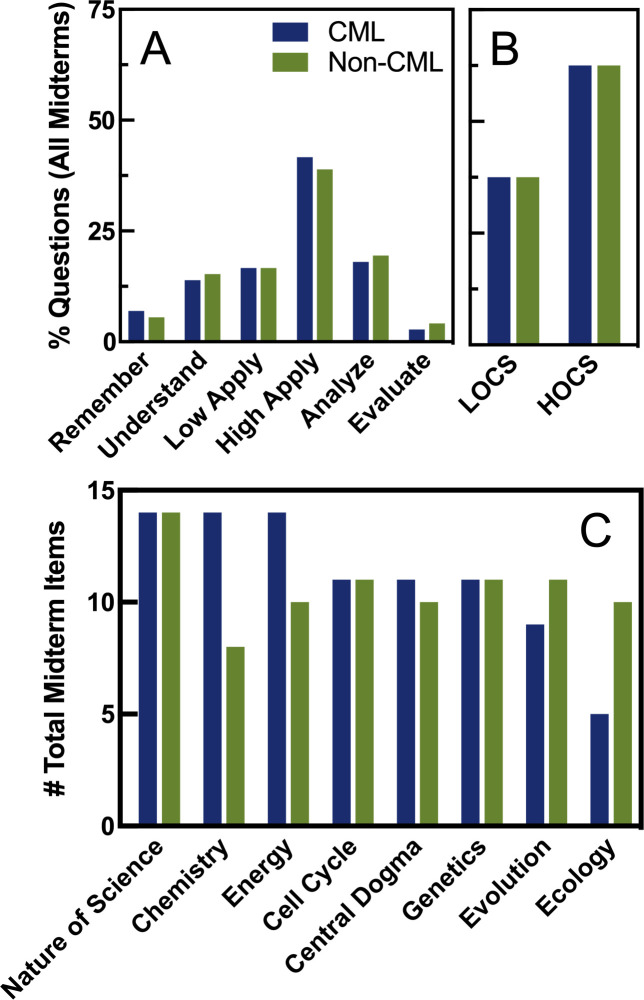
Midterms were equivalent in terms of cognitive skill, but topics could not be covered equally. Panel A: All cumulative (blue) and non-cumulative (green) midterm exam items were categorized by Bloom’s taxonomy, and percent of total is shown on the y axis. Counts were indistinguishable by section (*X*^*2*^(5, n = 144) = 0.46, *p* = 0.99). Panel B: All midterm exam items categorized by LOCS and HOCS are shown as a percentage of the whole on the y axis. Counts were indistinguishable by section (*X*^*2*^(1, n = 144) < 0.001, *p* > 0.99). Panel C: Total number of midterm items covering each course topic is shown for each section. Course topics are shown in the order in which they were covered in the course.

Because of our experimental question, we purposefully administered different midterm exams to the two sections. However, we only wanted the midterm exams to differ substantially in terms of content (current unit versus past material) while keeping them as similar as possible in terms of length (always 12 multiple-choice questions) and the cognitive skills required. All test items were classified using Bloom’s taxonomy [49–51] and LOCs/HOCS [50, 52] as described in the Methods. The average number of questions on each midterm requiring high-order cognitive skills was indistinguishable for the cumulative section (mean = 7.71, SD = 1.38, n = 7) and non-cumulative section (mean = 7.71, SD = 2.29, n = 7) by paired t-test (t(6) = 0; *p* > 0.99), with the majority of midterms containing six to nine high-level questions out of 12. If all midterm questions were compared as a whole, the cumulative and non-cumulative sections were near identical in terms of cognitive skills required across the semester (Fig 2A and 2B). The cumulative section did end up having more midterm questions about earlier content while the non-cumulative section had more midterm questions about later content (see Fig 2C), but this was unavoidable.

### Study habits

At the end of the semester, we asked students about their study habits to see if midterm format (cumulative or non-cumulative) had an effect on the amount they studied per week during the semester. Weekly study hours for students who took cumulative midterms (mean = 3.33 hours per week, SD = 1.77, n = 149) and those who took non-cumulative midterms (mean = 3.57 hours per week, SD = 1.86, n = 148) were indistinguishable by independent samples t-test (t(295) = 1.12; *p* = 0.26).

**Fig 3:**
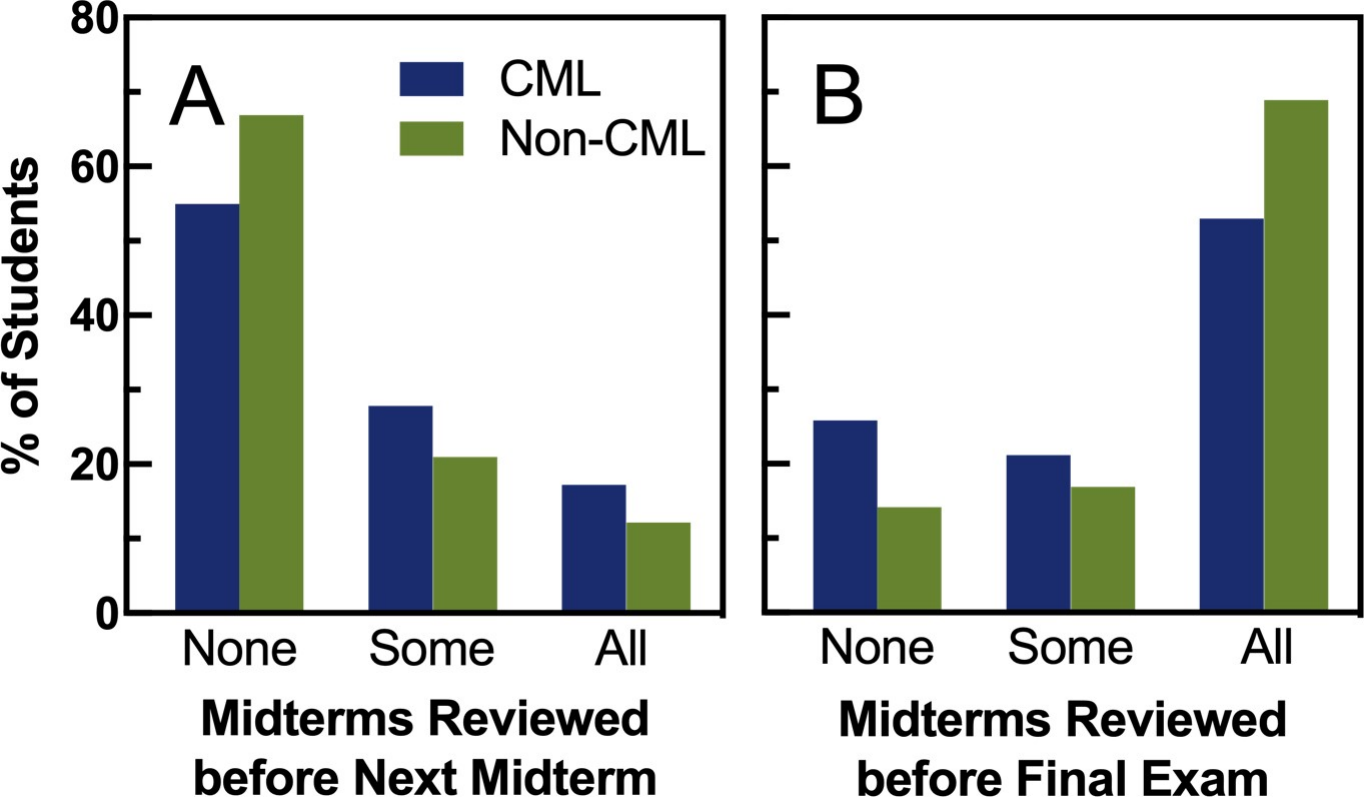
Effect of midterm type on timing of past exam review. In at attitudinal survey at the end of the semester, students self-reported the number (none, some, or all) of midterm exams that they reviewed prior to the next midterm exam (Panel A; Mann-Whitney U test, U = 12531.5, *p* = 0.037, n = 299), and in preparation for the final exam (Panel B; Mann-Whitney U test, U = 9241.5, *p* = 0.003, n = 299).

Since students had access to all midterm exams and their keys immediately after the tests closed, we also asked students how many (none, some, all) of those midterm exams they reviewed before the next midterm or in preparation for the final exam. As shown in Fig 3, most students used the midterm exams as a study tool right before the final exam rather than throughout the semester regardless of midterm format. However, there were small differences between sections: students with cumulative midterms were more likely to study a past midterm before the next midterm (Fig 3A, *p* = 0.037) and students with non-cumulative midterms were more likely to study the midterms in preparation for the final exam (Fig 3B, *p* = 0.003). The quality of their midterm exam review (see Methods) did not differ by section (Mann-Whitney U test, U = 10199.5, *p* = 0.259, n = 296).

### Performance on exams

**Fig 4:**
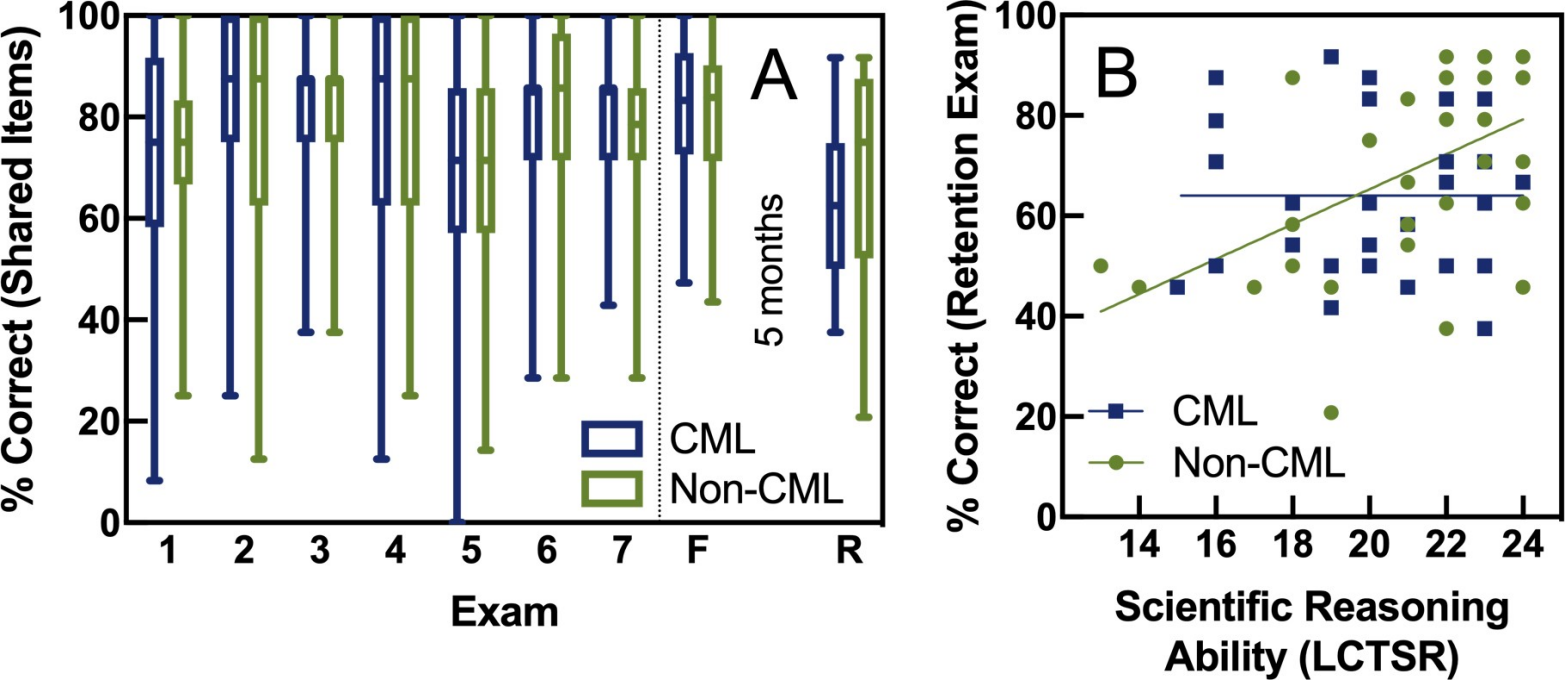
Effect of cumulative midterms on exam scores. Panel A: The y axis shows percent correct for midterms one through seven (calculated using only test items that were shared between cumulative, blue, and non-cumulative, green, sections), the final exam (F), and the retention exam (R) five months after the semester ended. Panel B: Retention exam scores are shown on the y axis and LCTSR scores are on the x axis. Lines are from a simple linear regression and show different relationships between scientific reasoning and retention for the non-cumulative (slope = 3.48, R^2^ = 0.27, *p* = 0.003) and cumulative (slope = -0.001, R^2^ = 0.00, *p* = 0.99) sections.

Student learning was assessed by performance on nine exams: seven midterms (only shared items were compared), a cumulative final exam (identical for both sections), and a retention exam five months after the final (identical for both sections). Shared items on midterm exams consisted mostly of questions about the most recent topic in the course, but all midterms also included one or two shared items about the nature of science and drawing conclusions from experiments. We first used a repeated measures analysis of variance (ANOVA; with Greenhouse-Geisser correction) to investigate the effect of midterm exam format on midterm and final exam scores. This analysis did not allow for missing data, so only students who took all midterms and the final exam were included. As shown in Fig 4A, we saw no difference in midterm and final exam performance between the two sections (*F*(1, 299) = 0.003, *p* = 0.95; n = 301) nor an interaction between midterm type and exam (*F*(5.7, 1705) = 0.92, *p* = 0.47; n = 301). We analyzed scores for the retention exam using an independent samples t-test to determine the effect of treatment (midterm type). Again, Fig 4A shows no statistical difference in retention between the section that had cumulative midterms and the section that had non-cumulative midterms (*t*(1, 60) = 1.16, *p* = 0.25; n = 62).

In order to consider more student characteristics simultaneously, we used multiple linear regression with backward stepwise selection to target student performance on shared midterm items and the final exam. Final models are shown in Table 2 and full model selection is available in the S3, S4 Tables. Possible predictors included midterm format (cumulative versus non-cumulative), student gender, average number of hours studied per week, scientific reasoning ability at the beginning of the semester (LCTSR score), preparation (the percent of preparatory class assignments or activities completed), reviewed last midterm before the next (all, some, or none), reviewed midterms before the final (all, some, or none; not included when targeting shared midterm items), quality of midterm review, score on the final exam (only included when targeting the retention exam), and an interaction between midterm format and LCTSR score. As shown in Table 2, scores on shared midterm items and the final exam were predicted by preparation and scientific reasoning ability (as expected), but midterm format had no effect.

**Table 2:** Final results multiple linear regression to target student performance (% correct) on exams.

Target	R^2^	Adj. R^2^	F(df)	Variable	B	SE_B_	β	*p* value
Shared Midterm Items	0.306	0.301	F(2, 257) = 56.67	(Intercept)	27.022	5.642		<0.0005
				Preparation ^a^	0.221	0.050	0.232	<0.0005
				Scientific Reasoning ^b^	1.595	0.159	0.525	<0.0005
Final Exam	0.362	0.358	F(2,289) = 81.99	(Intercept)	20.945	5.456		<0.0005
				Preparation ^a^	0.288	0.048	0.282	<0.0005
				Scientific Reasoning ^b^	1.816	0.153	0.558	<0.0005
Retention Exam	0.572	0.542	F(4,56) = 18.73	(Intercept)	4.477	12.53		0.722
				Final Exam Score ^c^	0.823	0.115	0.696	<0.0005
				Cumulative*Reasoning	-3.454	1.130	-0.340	0.003
				Scientific Reasoning ^b^	-0.346	0.634	-0.055	0.588
				Cumulative Midterms	0.580	3.794	0.017	0.879

^a^ Preparation = % of preparatory class assignments and activities completed (included those that targeted the same learning objectives as the midterms: class attendance, reading assignments, homework assignments)

^b^ Assessed at the beginning of the semester using Lawson’s classroom test of scientific reasoning

^c^ The retention exam consisted of 24 questions modeled after 24 out of the 48 final exam items. The final exam score used here was a student’s score on just those 24 items.

We also used multiple linear regression to target long-term retention. With such a small sample size for the retention exam, only midterm format (cumulative versus non-cumulative), final exam score, scientific reasoning at the beginning of the semester (LCTSR score), and an interaction between midterm format and LCTSR score were included as predictors to target retention. As shown in Table 2, final exam score was a significant predictor as expected, and so was an interaction between midterm format and scientific reasoning ability (interaction shown visually with raw data in Fig 4B). For students who took non-cumulative exams (Fig 4B, green), there was a strong relationship between reasoning ability at the beginning of the semester and how much they retained five months after the final exam. However, there was no relationship between reasoning ability and retention for students who took cumulative exams (blue).

Due to the limited number of test items, not all learning objectives could be tested on each exam. Thus, the cumulative section only received additional practice on a subset of the learning objectives. Therefore, we wanted to test whether cumulative midterms had a greater impact if we only included final exam questions and retention exam questions that evaluated those learning objectives on which students got extra retrieval practice (tested on more than one cumulative midterm, excluding the nature of science; see S1 Fig). We repeated the multiple regressions of Table 2 but targeted these adjusted final exam and retention exam scores instead of the full scores. Results were practically identical to those shown in Table 2, with the same significant predictors (preparation and scientific reasoning for the final exam; final exam and an interaction between midterm format and reasoning for the retention exam) in the final models. Although not identical, coefficients were practically equivalent, so full regression results are not shown.

### Performance by cognitive skills

**Fig 5:**
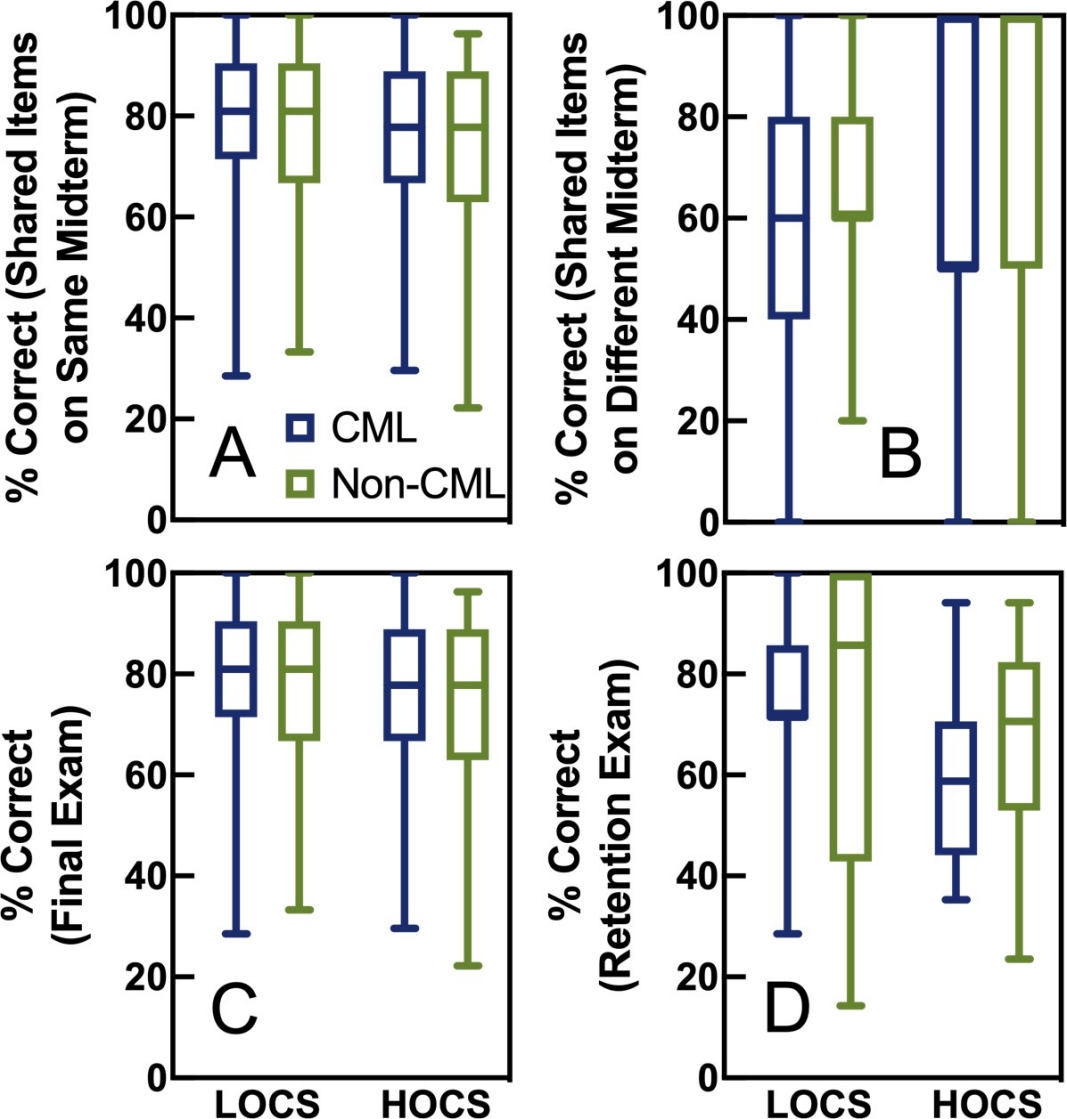
Effect of cumulative midterms by cognitive skill. All test items were categorized as requiring LOCS or HOCS and performance is shown by treatment (cumulative midterms = blue, non-cumulative midterms = green). The y axes show scores on shared midterm items that both sections saw the same day (Panel A, n = 344), shared midterm items that the cumulative section saw later (Panel B, n = 310), the final exam (Panel C, n = 344), and the retention exam (Panel D, n = 62).

Next, we wondered if cumulative midterms would influence student performance on items of varying cognitive complexity. Repeated measures ANOVAs were used to determine the effect of treatment (midterm format) on student performance on LOCS versus HOCS questions. As shown in Fig 5A, midterm format had no effect on shared midterm items (*F*(1, 289) = 0.012, *p* = 091), and there was no interaction between midterm format and cognitive level (*F*(1, 289) = 0.87, *p* = 0.35). There were a few questions that both sections completed, but the cumulative section saw them on a later midterm exam compared to the non-cumulative section. As shown in Fig 5B, the students who were given the test items right after learning the material (i.e. those in the non-cumulative section) earned higher scores than students in the cumulative section who were tested after some time had passed (*F*(1, 308) = 15.82, *p* < 0.0005, partial η^2^ = 0.05), and this was especially true for high-order questions (interaction between level and midterm type: *F*(1, 308) = 7.93, *p* = 0.005, partial η^2^ = 0.03).

Final exam scores by cognitive level are shown in Fig 5C: midterm format had no overall effect on final exam scores (*F*(1, 342) = 0.27, *p* = 0.61), but there was a hint of an interaction between midterm format and cognitive level (*F*(1, 342) = 3.41, *p* = 0.07, partial η^2^ = 0.01). Students who took cumulative exams may have performed better on low-order questions on the final exam, but if real, it was only a small effect. Five months later on the retention exam, midterm format had no overall effect on scores (*F*(1, 60) = 0.71, *p* = 0.40), but there was a medium-sized interaction between midterm format and cognitive level (*F*(1, 60) = 4.08, *p* = 0.048, partial η^2^ = 0.06). Fig 5D shows this interaction: students who took non-cumulative midterms performed better on high-order questions compared to students who took cumulative midterms, but performance on low-order questions was relatively equivalent.

### Performance by topic

**Fig 6:**
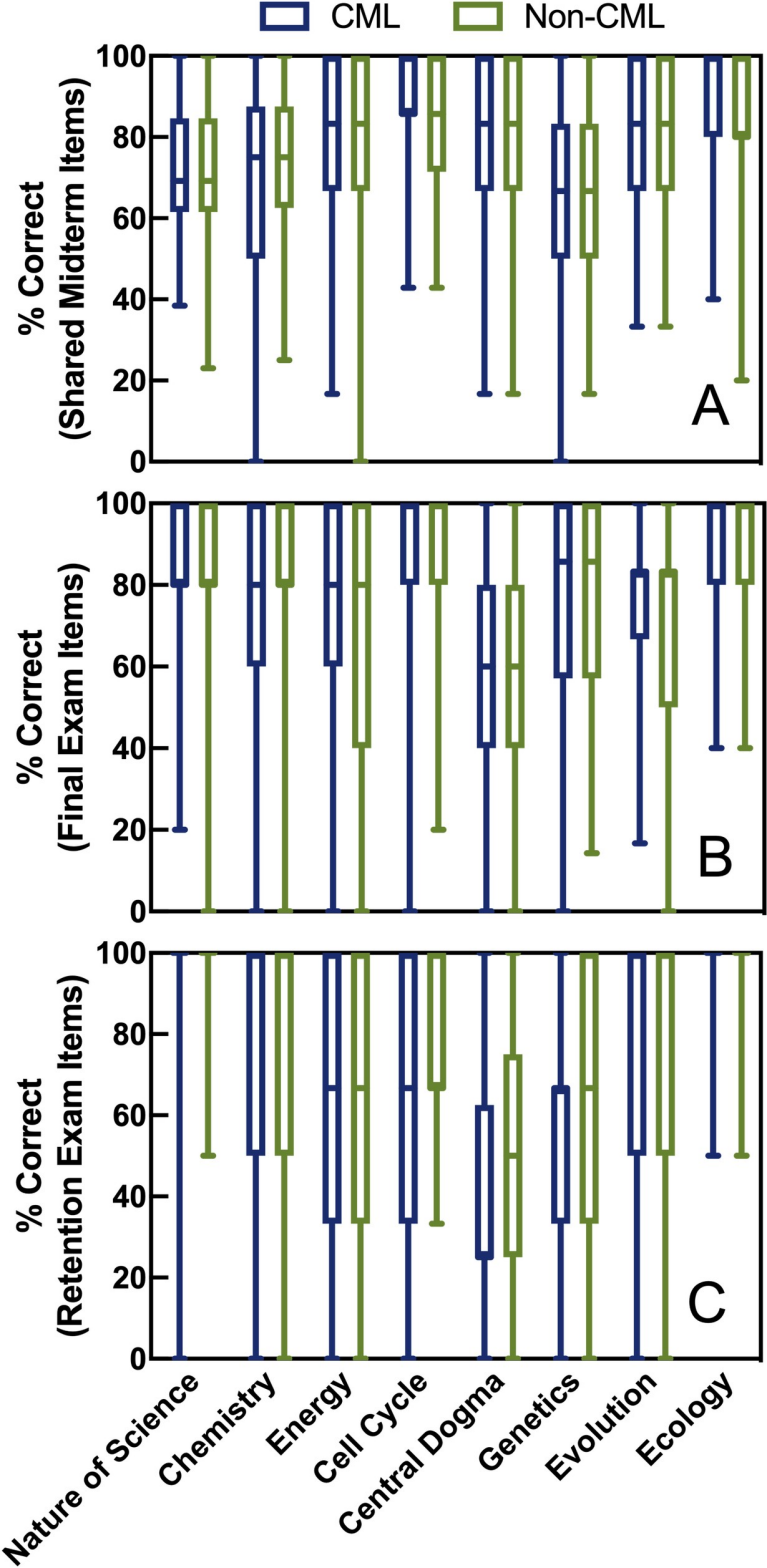
Effect of cumulative midterms on early versus late course topics. Student performance on shared midterm items (Panel A), final exam (Panel B), and retention exam (Panel C) are shown on the y axes by treatment (blue: cumulative midterms, green: non-cumulative midterms). Scores are grouped by topic, with topics displayed in the order in which they were covered in the course.

Finally, we wondered if cumulative midterms would influence student learning depending on the topic (early units versus late units). We again used repeated measures ANOVAs with Greenhouse-Geisser correction to compare section performance by topic. For shared midterm items (Fig 6A), we only included students who took every midterm. We found no effect of midterm format (*F*(1,299) = 0.002, *p* = 0.97, n = 301) nor an interaction between midterm format and topic (*F*(6.4,1908) = 1.016, *p* = 0.42). For final exam scores (Fig 6B), we also found no significant effect of midterm format (*F*(1,342) = 0.223, *p* = 0.64, n = 344) nor an interaction between midterm format and topic (*F*(6.2,2112) = 1.523, *p* = 0.16). Similarly, midterm format had no effect on retention exam scores (Fig 6C; *F*(1,60) = 1.665, *p* = 0.20, n = 62), and there was no interaction between midterm format and topic (*F*(5.8,346) = 0.656, *p* = 0.68).

To test this more thoroughly, we performed linear mixed model regression so we could account for students’ preparation for each topic and investigate a possible interaction between midterm format and topic order (early versus late topics). We excluded scores on the nature of science topic in this analysis since the two sections technically did not receive different treatments in terms of that topic. Detailed results from the random effect and fixed effect selection processes are available in the S5–S10 Tables. As shown in Table 3, topic-specific preparation (completing course assignments and activities that targeted the topic’s learning objectives) and scientific reasoning ability were retained in the final models to predict student performance on both the shared midterm items and the final exam. Since the maximum number of preparation assignments/activities for each topic was 4, a student who did all of the preparation was predicted to score 20% higher (regression coefficient ~ 5, Table 3) on that topic on the midterms and 12% higher (regression coefficient ~ 3, Table 3) on the final than a student who did none. An interaction between midterm format and the topic order was also retained in the best model to predict shared midterm item scores, but this interaction was the opposite we hypothesized. We had hypothesized that cumulative midterms would benefit students on early topics, since they would get the most retrieval practice on those topics, and that non-cumulative midterms might benefit students on later topics as they would have more time to devote to the later topics than their cumulative midterm peers. However, the positive coefficient for the interaction in Table 3 suggested that students who had cumulative midterms did better than students who took non-cumulative midterms, but only on later topics. The effect size of this interaction was very small, with each later topic giving the students with cumulative midterms an advantage of less than 1% (regression coefficient ~ 0.7, Table 3), and this interaction between topic order and midterm format was not retained in the best model to predict student scores by topic on the final exam (Table 3).

**Table 3:** Final linear mixed models to predict performance on shared midterm items, final exam, and retention exam by topic.

Target	Parameter^a^	Relative Variable Importance	Included in best model^b^?	Regression Coefficient ± SE
Shared Midterm Items by Topic	(Intercept)			28.845 ± 4.756
	Topic Preparation	1.00	Yes	5.056 ± 0.669
	Scientific Reasoning	1.00	Yes	1.671 ± 0.151
	Cumulative*Topic.Order	0.83	Yes	0.667 ± 0.289
	Cumulative*Reasoning	0.35	No	
	Cumulative Midterms	0.27	No	
Final Exam by Topic	(Intercept)			26.203 ± 5.600
	Topic Preparation	1.00	Yes	3.192 ± 0.756
	Scientific Reasoning	1.00	Yes	2.060 ± 0.186
	Cumulative*Topic.Order	0.46	No	
	Cumulative Midterms	0.33	No	
	Cumulative*Reasoning	0.28	No	
Retention Exam by Topic	(Intercept)			29.424 ± 7.041
	Final Exam Score	1.00	Yes	44.257 ± 5.952
	Cumulative*Reasoning	0.97	Yes	-3.739 ± 1.071
	Scientific Reasoning	0.43	No	
	Cumulative*Topic.Order	0.35	No	
	Cumulative Midterms	0.27	No	

^a^ Topic Preparation = completion of assignments that targeted that topic’s learning objectives, max = 4; Scientific Reasoning = score on LCTSR at the beginning of the class; Cumulative*Reasoning = interaction between midterm format and scientific reasoning; Cumulative*Topic.Order = interaction between midterm format and topic order (topics 2–7; topic 1 was not included since these questions did not differ between sections); Final Exam Score = score on 24 items corresponding to retention exam.

^b^ The best models also included random effects to allow for random intercepts for each student and topic: (1|Student) and (1|Topic)

We also used linear mixed models to predict scores by topic on the retention exam. Final exam scores were used as a predictor instead of topic preparation to control for student learning by the end of the semester. As expected, final exam score on that topic was retained in the best model to predict scores by topic on the retention exam (Table 3). After controlling for how they performed on the final, other variables retained in the final model would then explain how much the student did or did not retain compared to their original performance. The only other variable included in the best model was an interaction between midterm exam format and scientific reasoning ability. This interaction was the same as the one found to predict retention exam scores overall in Table 2 and shown visually in Fig 4B: students with low reasoning ability at the beginning of the semester retained more if they had cumulative midterms throughout the semester.

### Attitudes

Finally, we asked students about their attitudes regarding the midterms and their format at the end of the semester. Students in the cumulative midterm and non-cumulative midterm sections were indistinguishable in their overall attitude toward the midterm exams (Mann-Whitney U test: U = 11273, *p* = 0.81, n = 298; overall mean = 3.3 out of 5-point Likert scale with 5 = very favorable) and belief that the time spent on the midterm exams helped them learn (S2A Fig; Mann-Whitney U test: U = 11365, *p* = 0.70, n = 298). We then asked the students whether the midterm exams would have been more helpful if they had been the opposite format; i.e. we asked students in the cumulative section if non-cumulative exams would have been more helpful, and we asked students in the non-cumulative section if cumulative exams would have been more helpful. As shown in S2B Fig, students in the non-cumulative section were more likely to think the other treatment would have been more helpful (Mann-Whitney U test: U = 9273, *p* = 0.009, n = 299).

When some of the students returned for the retention exam five months later, we asked them which midterm format was likely to lead to greater retention of course material. The vast majority of students in both sections thought that cumulative midterm exams would increase retention of course content (S2C Fig). However, the answer distributions did differ by section (Mann-Whitney U test: U = 627, *p* = 0.01, n = 61): the students who took cumulative midterms were more likely to say that cumulative midterms would increase retention by a lot, and students who took non-cumulative midterms were more likely to say that cumulative midterms would just increase retention by a little. We also asked what type of midterm they had been given when they were in the course, and as shown in S2D Fig, students who took cumulative midterms were more likely to remember the treatment they had been given (Fisher’s exact test: *p* = 0.05, odds ratio = 3.39, n = 61).

## Discussion

### Effect of cumulative testing on retention

We originally hypothesized that frequent cumulative midterm exams would increase short-term retention compared to frequent non-cumulative midterms in an introductory biology course. However, as shown in Figs 4A, 5C and 6B and Tables 2 and 3, midterm type had no effect on final exam scores, not even on early topics on which the cumulative section had the most retrieval practice. Our findings contradict studies conducted in introductory psychology and mathematics classes which found short-term retention benefits on the final exam for cumulative midterms compared to non-cumulative [4, 5].

We did find some evidence to support our hypothesis that frequent cumulative testing increases long-term retention more than non-cumulative testing, although it was only supported for some students. Five months after the course ended, students’ retention was impacted by midterm format differently depending on how they scored on a measure of scientific reasoning at the beginning of the semester (see Fig 4B and Table 2). Students with average scientific reasoning skills performed equally regardless of treatment, students with lower levels of scientific reasoning appeared to retain more if they took cumulative midterms during the semester, and students with high reasoning skills scored higher if they took non-cumulative midterms (Fig 4B and Table 2). The coefficient for the interaction between cumulative midterms and reasoning from Table 2 suggests that cumulative midterms reduced retention scores by ~15% for students with the highest reasoning scores and increased retention scores by ~15% for students with the lowest reasoning scores. While this was five months after the final and thus not part of the course grade, this is a huge difference equivalent to the difference between a D and a B–. Our results roughly match those found in a study done in an introductory psychology course that found greater benefits on retention from cumulative midterms for low-performing students compared to high-performing students two months after the course ended [5]. Our effect size was larger, but our retention period was also 2.5 times as long. We must also acknowledge that our subset of students who returned were more likely to be higher reasoners compared to those who did not return. Thus, it is possible that the interaction between treatment and reasoning ability could have been even larger had the lowest reasoners returned to take the long-term retention exam.

### Why not retention benefits for all? Context matters

We had confidently hypothesized that cumulative testing would increase retention for all students, since extra retrieval practice and encouraging spaced study of material are such well accepted principals of effective learning. Yet, we saw no benefits of cumulative testing for any students on the final exam, and it may actually have been detrimental for students with high reasoning ability. We consider some possible explanations below.

First, Nguyen and McDaniel [54] suggest that simply adding exams is not sufficient for increased retention; rather, instructors need to engineer quizzes to target the same learning objectives repeatedly for students to experience the testing effect. The cumulative section got additional retrieval opportunities for some learning objectives, but not all. We repeated the regression analyses of Table 2 and targeted only those final exam and retention exam questions on which cumulative exam students had been given extra practice (more than one midterm included a question on that learning objective), but the results of Table 2 remained unchanged. Thus, we do not see evidence of the testing effect even when defining related material very narrowly. However, it is possible that the short length of the midterms did not allow for the cumulative midterm section to have enough retrieval opportunities throughout the semester for enough specific learning objectives. As described in the Methods section, there were only 12 questions on each midterm, half of the cumulative section’s exam items tested current material, and one or two exam items tested the nature of science for both sections. Thus, only four or five unique questions remained to test old material on each midterm for the cumulative section. In a previous study, we found that some learning objectives targeting higher Bloom’s levels (e.g. analyze) required six testing opportunities before student performance plateaued [8]. While some nature of science learning objectives were tested four to six times (see S1 Fig), this was true for both sections. Aside from that content, the biggest benefit cumulative midterm students saw on a specific learning objective was an additional one to three retrieval opportunities throughout the semester (S1 Fig), which may not have been enough.

On a related note, benefits of cumulative testing and the number of retrieval opportunities needed likely depend on the cognitive tasks emphasized. Our assessments contained about 60% questions requiring HOCS (Fig 2B), and past studies have reached conflicting conclusions regarding whether the testing effect is relevant for HOCS [8, 37, 55]. Neither Lawrence nor Beagley and Capaldi, two studies that have reported final exam gains after cumulative midterms, provide information about the cognitive tasks required of their students [4, 5], but it is possible that past studies used exams that focused more on LOCS like memorization. Our data show that cumulative midterms may have benefited students on LOCS final exam questions slightly more than HOCS questions (Fig 5C) which could suggest that we may have seen greater benefits from cumulative testing had more of our test items required only LOCS. Interestingly, we saw an interaction between midterm type and cognitive skills on the long-term retention exam (Fig 5D), with cumulative midterms providing a slight benefit on low-level items and non-cumulative midterms actually increasing retention on high-level questions. This was surprising to us, but students in the non-cumulative section still got the same amount of practice on higher-order cognitive skills in general (Fig 2A and 2B) even if they did not get as many practice opportunities on specific learning objectives (S1 Fig). Perhaps these students benefitted from repeated practice on HOCS applied to different content, facilitating better transfer than students who saw the HOCS applied to specific content repeatedly. However, this contradicts results from a previous study that suggested the skill and the content must be retrieved together for the testing effect to influence learning of high-level skills [37].

Non-cumulative students also had practice retrieving skills and content together on homework assignments, and studies have found that the testing effect might not have additional benefit in courses that already contain learning activities that use similar processes of generation, retrieval, and application [54, 56]. The course used in this study included two assignments before midterm exams that gave students retrieval and application practice: (1) students completed practice questions targeting the same learning objectives as midterm exam items, graded their work, and wrote down what they learned from each question they missed; (2) students verbally taught a peer about the learning objectives from memory and then asked that peer questions as they taught about the same learning objectives. Both sections completed these assignments prior to each midterm. Thus, this course may have already provided enough opportunities for students to practice retrieval, and more testing opportunities may have been redundant.

Another way our course structure differed from past studies relates to the frequency of assessment. Previous studies only employed three midterms [4, 5], so our students received more than double the testing opportunities with seven midterms throughout the semester. Because studies have suggested that increasing the number of exams increases student learning [8, 11], it is possible that the benefits of frequent testing, present in both sections, already increased short-term retention close to the limit of what is achievable.

We had hypothesized that cumulative testing would increase retention of material due to increased study spacing on past material, but encouraging that spaced study through course structure may not always result in actual changes in student study habits. We did see a hint that students in the cumulative section reviewed past material more during the semester and crammed less before the final exam (Fig 3). However, most students in both sections still did most of their review right before the final exam (Fig 3B), so midterm type may not have had as large an impact on study habits as we had hoped. We also saw no difference in the self-reported average number of hours per week studied between the cumulative section and the non-cumulative section. It is possible that our creative grading scheme [8] that allowed midterm exams to be dropped, decreasing the stakes, could have influenced students to not adjust their study habits as much as they would have for fewer, higher-stakes midterm exams. Another study did report that students were more likely to space their study for exams worth a lot of points in the course [45]. For example, a study conducted with medical students employed only three assessments during the semester that together composed their final course grade. They found that students who were in the cumulative testing treatment self-reported 69 more hours of studying done throughout the semester than the control group [10]. These results differed from ours drastically, which could be attributed to the higher stakes the medical school students faced while taking the midterm exams. They also may have found a greater difference in study hours since their control group only took one exam at the end of the semester while our non-cumulative students took exams repeatedly. Although our students were tested on different material, students in both sections were encouraged to prepare for an exam frequently, which may have encouraged them to space their study throughout the semester regardless of the treatment.

Because students in both sections reported spending the same number of hours studying on average, perhaps students in the non-cumulative section spent more of that time studying current material since they did not have to review past material for the upcoming exam. However, if students in the non-cumulative section had more time to spend on current material, we would predict an interaction between cumulative testing and topic order since students in the cumulative section would have more past material to review as the semester progressed. As shown in Table 3 and Fig 6C, there was no significant interaction between cumulative testing and topic order on short- or long-term retention. Interestingly, there was a significant interaction between cumulative testing and topic order on shared midterm items (Table 3 and Fig 6A) but in the opposite direction as predicted: students in the cumulative section outperformed the non-cumulative section more on later topics when they had more past material to review. Furthermore, study habits were included as possible predictors in all models of Table 2 but were never retained in the final models. Thus, we do not have evidence that differential study habits impacted retention. We cannot dismiss the possibility that cumulative testing may still have impacted study habits in important ways that were not measured here, since students in the cumulative section were more likely to remember that their midterms were cumulative five months after the course ended (S2D Fig). In future research, we could gather more detailed data about weekly study hours and time spent on current content versus past content.

We are unsure why cumulative testing would have such different long-term retention effects on low- versus high reasoners (Fig 4B). We considered whether this interaction was related to performance on LOCS items versus HOCS items. If non-cumulative students spent more time studying current material, they may have employed deeper study strategies that better allowed them to master HOCS specifically (Fig 5D, [57, 58]). Perhaps the interaction seen in Fig 4B could be explained if non-cumulative exams allowed for more focused study that benefited students on HOCS items and that students with better reasoning skills were more experienced with deeper study strategies. To investigate this, we repeated the analysis of Table 2 but modeled the long-term retention of LOCS or HOCS individually as opposed to combined. As shown in S3 Fig, we found the same interaction between cumulative tests and scientific reasoning score as shown in Table 2 and Fig 4B. Thus, the differential benefit of cumulative testing based on reasoning score seems to apply to both LOCS and HOCS. Future research will be needed to (1) verify that frequent non-cumulative testing is truly better for high reasoners compared to frequent cumulative testing and (2) further investigate possible causal mechanisms. In addition, repeating this type of study in courses that emphasize standards-based assessment would be valuable to investigate whether cumulative midterms could help lower-performing students rise to meet standards without causing high-performing students to fall below expected standards.

## Limitations

Our study is first limited due to its quasi-experimental design. Since students choose their classes and choose in which sections of those classes they would like to enroll, we did not have the ability to randomly assign students into two treatments. In the regression analyses presented in this study, we do try to account for the impacts of uncontrolled variables between groups, but it is always possible that group differences could explain our results rather than our treatments. One novel contribution of our study is the investigation of differential effects of cumulative exams on test items requiring LOCS versus HOCS. However, a weakness of our study is that we did not purposefully choose the distribution of LOCS and HOCS on the exams with a priori predictions in mind. Thus, the interactions seen in Fig 5 may have been fortuitous. Finally, all long-term retention results should be interpreted cautiously, as only 62 of over 300 students returned to take the retention exam five months after the end of the course. That small sample size limits our statistical power to detect small effects and could exaggerate the observed effect size of the interaction between scientific reasoning and cumulative testing (Fig 4B). Furthermore, as show in S2 Table, higher reasoners were more likely to return for that test, so our conclusions are limited to that sample. Future research is needed to test long-term retention with larger and more complete student samples.

## Summary and implications

While the benefits of the testing effect and spaced study are demonstrated in laboratory studies, we continue to investigate the best ways to apply these learning strategies in the complicated ecosystem of a college course. Using cumulative testing, where midterm exams continually test students on all material that has been covered so far, is a logical way to provide repeated retrieval practice and encourage spaced study for students, yet very few studies have actually tested its effectiveness in classrooms. We propose that the benefits of cumulative midterms likely depend on the type of students enrolled in the course and other course characteristics.

Here, we tested the benefits of cumulative midterms compared to non-cumulative midterms in an introductory biology course that already encouraged spaced study by testing frequently (seven midterms throughout the semester), included a grading scheme that allowed midterm exams to be either high stakes or low stakes (depending on what benefited the student), provided retrieval practice on homework assignments before each midterm, and emphasized high-level cognitive skills. We found that in this context, cumulative midterms provided no short-term benefit, as students in both treatments earned equivalent scores on the shared cumulative final exam (Figs 4A, 5C and 6B). Cumulative midterms did appear to increase long-term retention five months after the course ended, but this was only true for students who entered the course with low levels of scientific reasoning ability (Fig 4B). In fact, cumulative midterms may have decreased long-term retention for high-reasoning students. Thus, we propose that cumulative midterms may not be needed in courses that already provide frequent retrieval opportunities and encourage spaced study and/or in courses with high-performing students. However, in courses that include less-prepared students, cumulative midterms may help level the playing field.

## Supporting information

S1 Table Cumulative and non-cumulative sections were generally equivalent in the returning sample who took the retention exam. (PDF)

S2 Table High-scoring students and STEM majors were more likely to return for the retention exam. (PDF)

S3 Table Full model selection for Table 2, targeting performance on shared midterm items (backwards multiple linear regression). (PDF)

S4 Table Full model selection for Table 2, targeting performance on final exam (backwards multiple linear regression). (PDF)

S5 Table Selection of random effects to predict shared items on midterms by topic. (PDF)

S6 Table Selection of fixed effects to predict shared items on midterms by topic (top 10 models). (PDF)

S7 Table Selection of random effects to predict final exam by topic. (PDF)

S8 Table Selection of fixed effects to predict final exam by topic (top 10 models). (PDF)

S9 Table Selection of random effects to predict retention exam by topic. (PDF)

S10 Table Selection of fixed effects to predict retention exam by topic (top 10 models). (PDF)

S1 Fig Number of testing opportunities during the semester for each learning objective tested on the final exam by midterm type. (PDF)

S2 Fig Student attitudes regarding cumulative and non-cumulative midterms. (PDF)

S3 Fig Interaction between scientific reasoning and midterm type for long-term retention (LOCS and HOCS individually). (PDF)

S1 Appendix Examples of similar questions testing the same learning objective. (PDF)

S2 Appendix Examples of test question categorization. (PDF)

S1 Dataset (CSV)
