# Supplementary material for: The retention benefits of cumulative versus non-cumulative midterms in introductory biology may depend on students’ reasoning skills
Source: PLoS One. 2021 Apr 22;16(4):e0250143. doi: 10.1371/journal.pone.0250143 (PMC8062001; doi:10.1371/journal.pone.0250143)
Supplement: S1 Table — (PDF) [file pone.0250143.s001.pdf]

S1 Table. Cumulative and Non-cumulative sections were generally equivalent in the returning sample who took the retention exam.

Variable	Cumulative			Non-Cumulative			Statistical Test	*p*
Major [a]	12 STEM, 13 not			13 STEM, 15 not			Fisher's exact	0.99
Gender [a]	14 male, 15 female			19 male, 14 female			Fisher's exact	0.61
	**Mean**	**SD**	**N**	**Mean**	**SD**	**N**		
Scientific Reasoning [b]	20.21	2.61	29	21.06	2.84	32	Ind. samples t	0.23
Year in School [a]	2.00	1.12	25	1.64	0.62	28	Mann Whitney U	0.42
Pre-Interest in Bio [ac]	2.85	1.13	27	2.93	1.07	29	Mann Whitney U	0.92
Attendance [d]	97.39	5.62	29	95.96	7.71	33	Ind. samples t	0.41
Writing Assignments [d]	94.88	8.74	29	89.97	16.95	33	Welch's t	0.15
Reading Assignments [d]	88.10	18.55	29	91.20	10.74	33	Ind. samples t	0.42
Other Homework [d]	89.36	16.15	29	88.95	14.49	33	Ind. samples t	0.92
Final Exam Score [e]	83.48	13.80	29	82.20	15.63	33	Ind. samples t	0.74

[a] Self-reported

[b] Assessed at the beginning of the semester using Lawson's classroom test of scientific reasoning

[c] Data self-reported

[d] Scores are reported as percentage points earned by the end of the semester

[e] Only those items that were similar to retention exam items.
