# Supplementary material for: The retention benefits of cumulative versus non-cumulative midterms in introductory biology may depend on students’ reasoning skills
Source: PLoS One. 2021 Apr 22;16(4):e0250143. doi: 10.1371/journal.pone.0250143 (PMC8062001; doi:10.1371/journal.pone.0250143)
Supplement: S2 Table — (PDF) [file pone.0250143.s002.pdf]

S2 Table. High-scoring students and STEM majors were more likely to return for the retention exam.

Variable	Non-Returning			Returning			Statistical Test	*p*
Major [a]	86 STEM, 163 not			25 STEM, 28 not			Fisher's exact	0.09
Gender [a]	153 male, 130 female			33 male, 29 female			Fisher's exact	1.00
	**Mean**	**SD**	**N**	**Mean**	**SD**	**N**		
Scientific Reasoning [b]	18.30	4.09	278	20.66	2.74	61	Ind. samples t	<0.0005
Year in School [a]	1.51	0.80	248	1.81	0.90	53	Mann Whitney U	0.007
Pre-Interest in Bio [ac]	2.82	1.06	244	2.89	1.09	56	Mann Whitney U	0.58
Attendance [d]	97.00	7.67	283	96.63	6.80	62	Ind. samples t	0.73
Writing Assignments [d]	90.65	14.05	283	92.27	13.85	62	Ind. samples t	0.41
Reading Assignments [d]	91.04	12.99	283	89.75	14.86	62	Ind. samples t	0.49
Other Homework [d]	88.71	17.28	283	89.14	15.16	62	Ind. samples t	0.86
Final Exam Score	79.66	13.16	283	86.53	10.31	62	Welch's t	<0.0005

[a] Self-reported

[b] Assessed at the beginning of the semester using Lawson's classroom test of scientific reasoning

[c] Data self-reported

[d] Scores are reported as percentage points earned by the end of the semester
