# Supplementary material for: The retention benefits of cumulative versus non-cumulative midterms in introductory biology may depend on students’ reasoning skills
Source: PLoS One. 2021 Apr 22;16(4):e0250143. doi: 10.1371/journal.pone.0250143 (PMC8062001; doi:10.1371/journal.pone.0250143)
Supplement: S3 Table — (PDF) [file pone.0250143.s003.pdf]

S3 Table. Full model selection for Table 2, targeting performance on shared midterm items (backwards multiple linear regression).

Model	R^2	Adj. R^2	Significance (ΔR^2)	Variable	B	SE_B	β	*p* value
1	0.317	0.295	<0.0005	(Intercept)	30.679	6.075		<0.0005
				Cumulative Midterms	0.010	1.284	0.000	0.994
				Female	-0.836	1.350	-0.034	0.536
				Preparation	0.237	0.052	0.248	<0.0005
				Scientific Reasoning	1.517	0.175	0.499	<0.0005
				Review Quality	-0.555	0.722	-0.042	0.443
				Reviewed before Next [a]	-0.528	0.912	-0.032	0.564
				Hours Studied	-0.432	0.389	-0.065	0.267
				Cumulative*Reasoning	-0.126	0.322	-0.021	0.696
2	0.317	0.298	0.994	(Intercept)	30.686	5.989		<0.0005
				Female	-0.836	1.346	-0.034	0.535
				Preparation	0.237	0.052	0.248	<0.0005
				Scientific Reasoning	1.517	0.174	0.499	<0.0005
				Review Quality	-0.555	0.719	-0.042	0.441
				Reviewed before Next [a]	-0.526	0.901	-0.032	0.560
				Hours Studied	-0.432	0.386	-0.065	0.264
				Cumulative*Reasoning	-0.126	0.321	-0.021	0.696
3	0.316	0.300	0.696	(Intercept)	30.784	5.974		<0.0005
				Female	-0.844	1.344	-0.035	0.530
				Preparation	0.237	0.052	0.248	<0.0005
				Scientific Reasoning	1.511	0.173	0.497	<0.0005
				Review Quality	-0.529	0.714	-0.040	0.460
				Reviewed before Next [a]	-0.530	0.900	-0.032	0.557
				Hours Studied	-0.448	0.383	-0.067	0.243
4	0.315	0.302	0.557	(Intercept)	30.377	5.926		<0.0005
				Female	-0.898	1.339	-0.037	0.503
				Preparation	0.241	0.051	0.253	<0.0005
				Scientific Reasoning	1.516	0.173	0.498	<0.0005
				Review Quality	-0.568	0.710	-0.043	0.425
				Hours Studied	-0.505	0.371	-0.076	0.175
5	0.314	0.303	0.503	(Intercept)	29.888	5.875		<0.0005
				Preparation	0.236	0.051	0.247	<0.0005
				Scientific Reasoning	1.549	0.166	0.509	<0.0005
				Review Quality	-0.606	0.707	-0.045	0.392
				Hours Studied	-0.496	0.370	-0.074	0.182

6	0.312	0.304	0.392	(Intercept)	29.001	5.780		<0.0005
				Preparation	0.234	0.050	0.245	<0.0005
				Scientific Reasoning	1.530	0.164	0.503	<0.0005
				Hours Studied	-0.548	0.365	-0.082	0.134
7	0.306	0.301	0.134	(Intercept)	27.022	5.642		<0.0005
				Preparation	0.221	0.050	0.232	<0.0005
				Scientific Reasoning	1.595	0.159	0.525	<0.0005

[a] Reviewed the last midterm before the next (none, some, or all)
