# Supplementary material for: The retention benefits of cumulative versus non-cumulative midterms in introductory biology may depend on students’ reasoning skills
Source: PLoS One. 2021 Apr 22;16(4):e0250143. doi: 10.1371/journal.pone.0250143 (PMC8062001; doi:10.1371/journal.pone.0250143)
Supplement: S4 Table — (PDF) [file pone.0250143.s004.pdf]

S4 Table. Full model selection for Table 2, targeting performance on final exam (backwards multiple linear regression).

Model	R^2	Adj. R^2	Significance (ΔR^2)	Variable	B	SE_B	β	*p* value
1	0.367	0.346	<0.0005	(Intercept)	21.056	5.903		<0.0005
				Cumulative Midterms	0.694	1.267	0.027	0.584
				Female	-0.439	1.320	-0.017	0.739
				Preparation	0.300	0.051	0.293	<0.0005
				Scientific Reasoning	1.755	0.170	0.540	<0.0005
				Review Quality	0.496	0.709	0.035	0.485
				Reviewed before Next [a]	0.140	0.896	0.008	0.876
				Reviewed before Final [b]	-0.365	0.813	-0.023	0.654
				Hours Studied	-0.296	0.378	-0.041	0.434
				Cumulative*Reasoning	0.211	0.313	0.032	0.501
2	0.366	0.349	0.876	(Intercept)	21.137	5.870		<0.0005
				Cumulative Midterms	0.727	1.247	0.028	0.561
				Female	-0.428	1.316	-0.016	0.745
				Preparation	0.299	0.050	0.292	<0.0005
				Scientific Reasoning	1.753	0.170	0.539	<0.0005
				Review Quality	0.505	0.706	0.035	0.475
				Reviewed before Final [b]	-0.347	0.803	-0.021	0.666
				Hours Studied	-0.281	0.365	-0.039	0.442
				Cumulative*Reasoning	0.212	0.313	0.032	0.499
3	0.366	0.351	0.745	(Intercept)	20.906	5.818		<0.0005
				Cumulative Midterms	0.728	1.245	0.028	0.559
				Preparation	0.296	0.050	0.290	<0.0005
				Scientific Reasoning	1.770	0.162	0.544	<0.0005
				Review Quality	0.491	0.703	0.034	0.485
				Reviewed before Final [b]	-0.379	0.796	-0.023	0.635
				Hours Studied	-0.276	0.364	-0.039	0.449
				Cumulative*Reasoning	0.209	0.312	0.032	0.503
4	0.366	0.352	0.635	(Intercept)	20.777	5.804		<0.0005
				Cumulative Midterms	0.819	1.229	0.032	0.506
				Preparation	0.294	0.049	0.287	<0.0005
				Scientific Reasoning	1.768	0.162	0.544	<0.0005
				Review Quality	0.435	0.693	0.030	0.530
				Hours Studied	-0.284	0.363	-0.040	0.435
				Cumulative*Reasoning	0.218	0.311	0.033	0.485
5	0.365	0.354	0.53	(Intercept)	21.434	5.703		<0.0005
				Cumulative Midterms	0.767	1.225	0.030	0.532

				Preparation	0.295	0.049	0.289	<0.0005
				Scientific Reasoning	1.782	0.160	0.548	<0.0005
				Hours Studied	-0.245	0.357	-0.034	0.493
				Cumulative*Reasoning	0.200	0.310	0.031	0.519
6	0.364	0.355	0.532	(Intercept)	22.005	5.623		<0.0005
				Preparation	0.294	0.049	0.288	<0.0005
				Scientific Reasoning	1.781	0.160	0.547	<0.0005
				Hours Studied	-0.259	0.356	-0.036	0.468
				Cumulative*Reasoning	0.206	0.309	0.032	0.505
7	0.363	0.356	0.505	(Intercept)	21.795	5.609		<0.0005
				Preparation	0.294	0.049	0.288	<0.0005
				Scientific Reasoning	1.788	0.159	0.550	<0.0005
				Hours Studied	-0.236	0.354	-0.033	0.507
8	0.362	0.358	0.507	(Intercept)	20.945	5.456		<0.0005
				Preparation	0.288	0.048	0.282	<0.0005
				Scientific Reasoning	1.816	0.153	0.558	<0.0005

[a] Reviewed the last midterm before the next (none, some, or all)
[b] Reviewed the midterms in preparation for the final exam at the end of the semester (none, some, all)
