# Supplementary material for: The retention benefits of cumulative versus non-cumulative midterms in introductory biology may depend on students’ reasoning skills
Source: PLoS One. 2021 Apr 22;16(4):e0250143. doi: 10.1371/journal.pone.0250143 (PMC8062001; doi:10.1371/journal.pone.0250143)
Supplement: S5 Table — (PDF) [file pone.0250143.s005.pdf]

S5 Table. Selection of random effects to predict shared items on midterms by topic.

Rank	Model[a]	AICc	Δ*i*	Best Model[b]
1	(1|Student) + (1|Topic)	19854.07	0.00	*
2	(1|Topic)	20146.35	292.27	
3	(1|Student)	20223.26	369.19	
4	No random effects	20425.57	571.49	

[a] Although not shown, models include all fixed effects of interest: Cum + Prep + LCTSR + Cum*LCTSR + Cum*Topic.Order

[b] As described in Methods, if models were within 2 AICc, the model with the fewest number of parameters was chosen as the best model. Verified by calculating ICCs for Student (0.30) and Topic (0.11).
