# Supplementary material for: The retention benefits of cumulative versus non-cumulative midterms in introductory biology may depend on students’ reasoning skills
Source: PLoS One. 2021 Apr 22;16(4):e0250143. doi: 10.1371/journal.pone.0250143 (PMC8062001; doi:10.1371/journal.pone.0250143)
Supplement: S6 Table — (PDF) [file pone.0250143.s006.pdf]

**S6 Table. Selection of fixed effects to predict shared items on midterms by topic (top 10 models).**

| Rank | Model[a] | AICc | Δ*i* | $\omega_i$ | Best Model[b] |
|---|---|---|---|---|---|
| 1 | Prep + LCTSR + Cum*Topic.Order | 19866.9 | 0.00 | 0.40 | * |
| 2 | Prep + LCTSR + Cum*LCTSR + Cum*Topic.Order | 19868.1 | 1.21 | 0.22 | |
| 3 | Cum + Prep + LCTSR + Cum*Topic.Order | 19868.9 | 1.97 | 0.15 | |
| 4 | Cum + Prep + LCTSR + Cum*LCTSR + Cum*Topic.Order | 19870.1 | 3.18 | 0.08 | |
| 5 | Prep + LCTSR | 19870.2 | 3.32 | 0.08 | |
| 6 | Prep + LCTSR + Cum*LCTSR | 19871.4 | 4.52 | 0.04 | |
| 7 | Cum + Prep + LCTSR | 19872.2 | 5.28 | 0.03 | |
| 8 | Cum + Prep + LCTSR + Cum*LCTSR | 19873.4 | 6.49 | 0.02 | |
| 9 | LCTSR + Cum*Topic.Order | 19921.1 | 54.17 | 0.00 | |
| 10 | LCTSR + Cum*LCTSR + Cum*Topic.Order | 19922.3 | 55.38 | 0.00 | |

[a] Cum = Cumulative Midterms; Prep = Preparation (completion of assignments that targeted that topic's learning objectives, max = 4); LCTSR = Scientific Reasoning Ability at the beginning of the class; Cum*LCTSR = interaction between midterm format and reasoning; Cum*Topic.Order = interaction between midterm format and topic order. Although not shown, all models also included random effects to allow for random intercepts for each student and topic: (1|Student) and (1|Topic)

[b] As described in Methods, if models were within 2 AICc, the model with the fewest number of parameters was chosen as the best model.
