# Supplementary material for: The retention benefits of cumulative versus non-cumulative midterms in introductory biology may depend on students’ reasoning skills
Source: PLoS One. 2021 Apr 22;16(4):e0250143. doi: 10.1371/journal.pone.0250143 (PMC8062001; doi:10.1371/journal.pone.0250143)
Supplement: S8 Table — (PDF) [file pone.0250143.s008.pdf]

S8 Table. Selection of fixed effects to predict final exam by topic (top 10 models).

Rank	Model[a]	AICc	Δ*i*	ω_i	Best Model[b]
1	Prep + LCTSR	20843.9	0.0	0.26	*
2	Prep + LCTSR + Cum*Topic.Order	20844.2	0.3	0.22	
3	Cum + Prep + LCTSR	20845.3	1.4	0.13	
4	Cum + Prep + LCTSR + Cum*Topic.Order	20845.6	1.8	0.11	
5	Prep + LCTSR + Cum*LCTSR	20845.7	1.9	0.10	
6	Prep + LCTSR + Cum*LCTSR + Cum*Topic.Order	20846.1	2.2	0.09	
7	Cum + Prep + LCTSR + Cum*LCTSR	20847.2	3.3	0.05	
8	Cum + Prep + LCTSR + Cum*LCTSR + Cum*Topic.Order	20847.5	3.6	0.04	
9	LCTSR	20859.2	15.3	0.00	
10	LCTSR + Cum*Topic.Order	20859.7	15.8	0.00	

[a] Cum = Cumulative Midterms; Prep = Preparation (completion of assignments that targeted that topic's learning objectives, max = 4); LCTSR = Scientific Reasoning Ability at the beginning of the class; Cum*LCTSR = interaction between midterm format and reasoning; Cum*Topic.Order = interaction between midterm format and topic order. Although not shown, all models also included random effects to allow for random intercepts for each student and topic: (1|Student) and (1|Topic)

[b] As described in Methods, if models were within 2 AICc, the model with the fewest number of parameters was chosen as the best model.
