# Supplementary material for: The retention benefits of cumulative versus non-cumulative midterms in introductory biology may depend on students’ reasoning skills
Source: PLoS One. 2021 Apr 22;16(4):e0250143. doi: 10.1371/journal.pone.0250143 (PMC8062001; doi:10.1371/journal.pone.0250143)
Supplement: S9 Table — (PDF) [file pone.0250143.s009.pdf]

S9 Table. Selection of random effects to predict retention exam by topic.

Rank	Model[a]	AICc	Δ*i*	Best Model[b]
1	(1|Student) + (1|Topic)	4041.86	0.00	*
2	No random effects	4049.80	7.94	
3	(1|Topic)	4052.86	11.00	
4	(1|Student)	4091.92	50.06	

[a] Although not shown, models include all fixed effects of interest: Cum + Final + LCTSR + Cum*LCTSR + Cum*Topic.Order

[b] As described in Methods, if models were within 2 AICc, the model with the fewest number of parameters was chosen as the best model. Verified by calculating ICCs for Student (0.18) and Topic (0.17).
