# Supplementary material for: The retention benefits of cumulative versus non-cumulative midterms in introductory biology may depend on students’ reasoning skills
Source: PLoS One. 2021 Apr 22;16(4):e0250143. doi: 10.1371/journal.pone.0250143 (PMC8062001; doi:10.1371/journal.pone.0250143)
Supplement: S10 Table — (PDF) [file pone.0250143.s010.pdf]

S10 Table. Selection of fixed effects to predict retention exam by topic (top 10 models).

Rank	Model[a]	AICc	Δ*i*	ω_i	Best Model[b]
1	Final + Cum*LCTSR	4070.6	0.0	0.27	*
2	Final + LCTSR + Cum*LTSR	4071.2	0.6	0.19	
3	Final + Cum*LCTSR + Cum*Topic.Order	4071.8	1.3	0.14	
4	Final + LCTSR + Cum*LCTSR + Cum*Topic.Order	4072.4	1.9	0.10	
5	Cum + Final + Cum*LCTSR	4072.6	2.1	0.09	
6	Cum + Final + LCTSR + Cum*LCTSR	4073.3	2.7	0.07	
7	Cum + Final + Cum*LCTSR + Cum*Topic.Order	4073.9	3.3	0.05	
8	Cum + Final + LCTSR + Cum*LCTSR + Cum*Topic.Order	4074.1	3.5	0.05	
9	Cum + Final + LCTSR	4077.8	7.2	0.01	
10	Cum + Final	4078.4	7.8	0.01	

[a] Cum = Cumulative Midterms; Final = Final Exam Score (24 items corresponding to retention exam); LCTSR = Scientific Reasoning Ability at the beginning of the class; Cum*LCTSR = interaction between midterm format and reasoning; Cum*Topic.Order = interaction between midterm format and topic order. Although not shown, all models also included random effects to allow for random intercepts for each student and topic: (1|Student) and (1|Topic)

[b] As described in Methods, if models were within 2 AICc, the model with the fewest number of parameters was chosen as the best model.
