# Supplementary material for: The retention benefits of cumulative versus non-cumulative midterms in introductory biology may depend on students’ reasoning skills
Source: PLoS One. 2021 Apr 22;16(4):e0250143. doi: 10.1371/journal.pone.0250143 (PMC8062001; doi:10.1371/journal.pone.0250143)

S3 Figure. Interaction between scientific reasoning and midterm type for LOCS and HOCS individually.

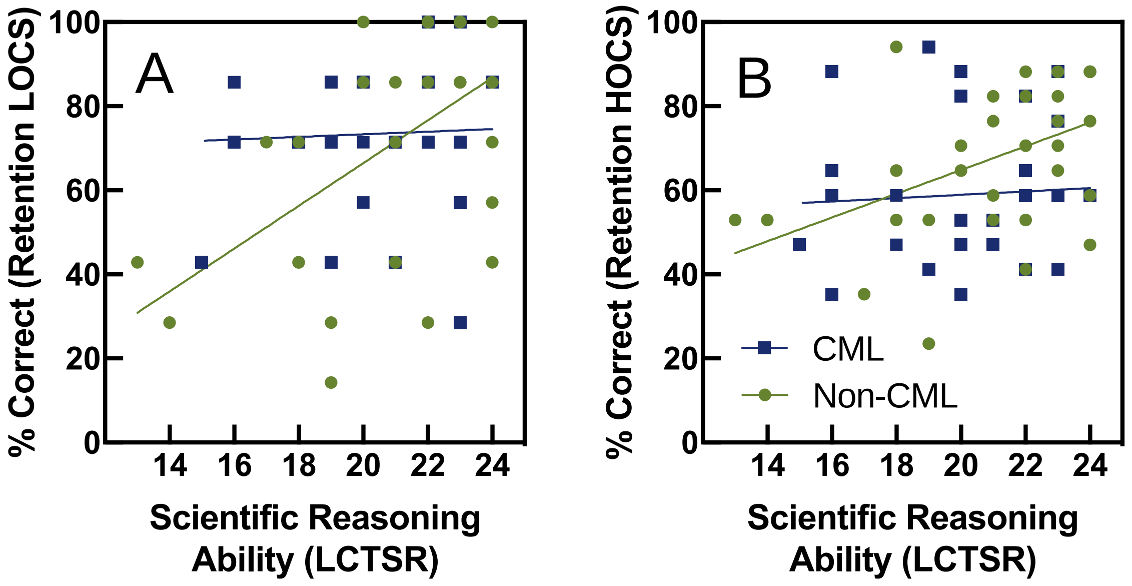

Supplement: S3 Fig — (PDF) [file pone.0250143.s013.pdf]
