# Supplementary material for: The retention benefits of cumulative versus non-cumulative midterms in introductory biology may depend on students’ reasoning skills
Source: PLoS One. 2021 Apr 22;16(4):e0250143. doi: 10.1371/journal.pone.0250143 (PMC8062001; doi:10.1371/journal.pone.0250143)
Supplement: S1 Appendix — (PDF) [file pone.0250143.s014.pdf]

S1 Appendix. Examples of similar questions testing the same learning objective.

GROUP 1

The ninhydrin test is used to detect individual amino acids that are not part of a polymer. The liquid turns blue-violet if single amino acids (not bonded to other amino acids) are present in the sample, and it remains colorless if no amino acids are present. The following ingredients were put into tubes as indicated and all chemical reactions were allowed to run to completion. Albumin is a protein, glycine is an amino acid, trypsin breaks down proteins into their monomers (but does not cut itself up), and amylase breaks down polysaccharides into mono- and di-saccharides. Which tubes would turn blue-violet after the ninhydrin test?

Tube	Contents
1	water
2	water, starch, albumin
3	water, starch, albumin, amylase
4	water, starch, albumin, trypsin
5	water, maltose, albumin
6	water, maltose, albumin, amylase
7	water, maltose, albumin, trypsin
8	water, maltose, glycine
9	water, maltose, glycine, amylase
10	water, maltose, glycine, trypsin
11	water, starch, glycine
12	water, starch, glycine, amylase
13	water, starch, glycine, trypsin

a. Tubes 2–13
b. Tubes 4, 7
c. Tubes 4, 8–13
d. Tubes 8–13
e. **Tubes 4, 7–13**
f. Tubes 4, 7, 10, 13
g. Tubes 3, 4, 6, 7, 8–13
h. Tubes 8, 9, 11, 12

Here is a table listing seven solutions placed in seven test tubes. Amylase is an enzyme in your saliva that breaks down polysaccharides into mono- and disaccharides. An element called iodine reacts specifically with starch molecules and thus is commonly used to detect starch. Iodine is usually yellowish-brown in color, but when it reacts with starch it turns purple. If I placed a drop of iodine into each of the tubes, which tubes would turn purple? Assume all reactions have time to run to completion.

Tube	Contents
1	Water
2	10% Cellulose solution
3	10% Cellulose + Amylase
4	10% Glucose solution
5	10% Starch solution
6	10% Glucose + Amylase
7	10% Starch + Amylase

a. tubes 2 and 3
b. tube 4
c. **tube 5**
d. tubes 4 and 5
e. tubes 4 and 7
f. tubes 5 and 7
g. tubes 5 and 6
h. tubes 6 and 7

Protein digestion begins in your stomach where an enzyme called pepsin breaks down proteins into its monomers. The Biuret test is sensitive to the presence of proteins and turns purple when they are present, but the solution is blue when they are absent. Ninhydrin solution turns purple in the presence of individual amino acids (not bonded to each other) and is clear otherwise. The ingredients in each of the following tubes were allowed to incubate for a sufficient amount of time for any reactions to go to completion, and then each tube was split into two parts so that both tests could be performed. Which tube's contents would turn blue after the Biuret test and purple after the ninhydrin test? (Although pepsin is a protein, you add the enzyme in such small amounts that its presence is not enough to affect the Biuret test.)

1	Water
2	albumin (a protein), water
3	glycine (an amino acid), water
4	albumin (a protein), water, pepsin
5	glycine (an amino acid), water, pepsin

a. Tube 2
b. Tube 3
c. Tubes 3 and 4
d. Tubes 2 and 4
e. Tubes 2 and 5
f. Tubes 3 and 5
g. **Tubes 3, 4, and 5**
h. Tubes 2, 3, 4, and 5

GROUP 2

If Protein L weighs approximately 37,200 g/mol, what is the best estimate for the weight of the coding region of *gene L* (the gene that codes for Protein L)? The average weight of a nucleotide is 310 g/mol. The average weight of an amino acid is 120 g/mol. All answers are in g/mol.

a. 310
b. 620
c. 930
d. 1,860
e. 43,200
f. 74,400
g. 86,400
h. 111,600
i. 288,300
j. **576,600**

If the coding region of the *RETQ gene* weighed 3,820,440 g/mol, how much would the RETQ protein weigh? The average weight of a nucleotide is 310 g/mol. The average weight of an amino acid is 120 g/mol.

a. 2,054 g/mol
b. 4,108 g/mol
c. 6,162 g/mol
d. 12,324 g/mol
e. **246,480 g/mol**
f. 492,960 g/mol
g. 1,273,480 g/mol
h. 1,910,220 g/mol

Gene X codes for a protein made of 327 amino acids. If I draw the mRNA made from *gene X* like this (showing only the start codon and the stop codon), how many nucleotides are missing in my drawing? (In other words, how many nucleotides do my dashed lines represent?)

5' AUG --> UAG 3'

a. 103
b. 106
c. 109
d. 975
e. **978**
f. 981
g. 1,950
h. 1,956
i. 1,962

GROUP 3

Scientists have found a high incidence of this allele in European and Mediterranean people. The frequency of the CCR5-Δ32 allele on the European continent is 10%. What percentage of the people are likely heterozygous for the CCR5 gene if the population is in Hardy-Weinberg equilibrium?

a. 90%
b. 81%
c. 10%
d. **18%**
e. 9%
f. 1%

The frequency of the recessive *GHRHR* allele that leads to Type I growth hormone deficiency is 0.002 in a population. If this population is in Hardy-Weinberg equilibrium, what is the frequency of Type I growth hormone deficiency?

a. **0.000004**
b. 0.002000
c. 0.003992
d. 0.003996
e. 0.044721
f. 0.912557
g. 0.996004
h. 0.998000

Huntington's disease is caused by a dominant allele. There is an abnormally high rate of this disease in the Lake Maracaibo region of Venezuela where 700 out of every 100,000 people are afflicted. What is the frequency of the dominant allele in that population? Assume Hardy-Weinberg equilibrium.

a. 0.0000123
b. **0.00351**
c. 0.00699
d. 0.993
e. 0.996

GROUP 4

Growth hormone deficiency can be caused by a variety of genetic mutations. Type I deficiency is caused by a recessive allele of the GHRHR gene, which is found on chromosome 7. Type II deficiency is caused by a dominant allele of the GH1 gene, which is found on chromosome 17. Type III deficiency is caused by a recessive allele of the BTK gene, which is found on the X chromosome. Bobby has growth hormone deficiency and so do his mother and his mother's mother. His father, both of his father's parents, and his mother's father are all normal. Can you diagnose Bobby with the correct deficiency type using these data? (Assume Bobby and his mother and his mother's mother all have the same type of growth hormone deficiency.)

a. Bobby has Type I deficiency.
b. Bobby has Type II deficiency.
c. Bobby has Type III deficiency.
d. **Bobby could have Type I or Type II deficiency.**
e. Bobby could have Type I or Type III deficiency.
f. Bobby could have Type II or Type III deficiency.
g. Bobby could have Type I, Type II, or Type III deficiency

You want to know what your blood type is, but you are deathly afraid of needles and don't want to be tested. You ask your parents and grandparents what their blood types are in order to figure it out yourself. Your mom is B+, and her parents are AB- and AB+. Your dad didn't know his blood type, but his parents are both O-. What is your blood type?

a. AB+
b. AB-
c. B+
d. B-
e. O+
f. O-
g. Could be A or B
h. **Could be C or D**
i. Could be E or F
j. Could be D or F

Color-blindness is an X-linked (meaning the gene is found on the X chromosome), recessive disorder. A color-blind boy has parents and grandparents that are all normal (none of them are color-blind). What are the genotypes of his mother and maternal grandmother (mother's mother)? We will use B for the dominant allele, and b for the color-blind, recessive allele.

a. Mom: X^BX^B Grandma: X^BX^B
b. Mom: X^BX^B Grandma: X^BX^b
c. Mom: X^BX^B Grandma: X^bX^b
d. Mom: X^BX^b Grandma: X^BX^B
e. **Mom: X^BX^b Grandma: X^BX^b**
f. Mom: X^BX^b Grandma: X^bX^b
g. Mom: X^bX^b Grandma: X^BX^B
h. Mom: X^bX^b Grandma: X^BX^b
i. Mom: X^bX^b Grandma: X^bX^b
j. There is not information to know one (or both) of their genotypes with confidence.

GROUP 5

Below is the original template strand sequence of a gene and then a mutant version of that same sequence. What type of mutation is this?

3' ACATGACACATACACCATGGTGCAGCTGCATCCGTAGTTCGTTATAT 5' (Original sequence)

3' ACATGACACATACACCAT**<u>C</u>**GTGCAGCTGCATCCGTAGTTCGTTATAT 5' (Mutated sequence)

a. **Nonsense**
b. Missense
c. Silent
d. Frameshift

If the sequence from the previous question (3' GATGTTACCAAGCATAGTATG 5') were mutated to the following, this would be what type of mutation?

3' GATGTTACCA**<u>G</u>**GCATAGTATG 5'

a. Missense
b. Nonsense
c. **Silent**
d. Frameshift
