# Supplementary material for: The retention benefits of cumulative versus non-cumulative midterms in introductory biology may depend on students’ reasoning skills
Source: PLoS One. 2021 Apr 22;16(4):e0250143. doi: 10.1371/journal.pone.0250143 (PMC8062001; doi:10.1371/journal.pone.0250143)
Supplement: S2 Appendix — (PDF) [file pone.0250143.s015.pdf]

S2 Appendix. Examples of test question categorization.

Cognitive Skill Level	Bloom's Level	Example Question
Low-order cognitive skills (LOCS)	Remember	Which of the following statements comparing prokaryotes and eukaryotes is true?<br>a. **Eukaryotic cells have a nucleus surrounded by a nuclear membrane; prokaryotic cells don't.**<br>b. Eukaryotic cells don't have cell walls; many prokaryotic cells do.<br>c. The genetic material of eukaryotic cells is DNA; the genetic material of prokaryotic cells can be either RNA or DNA.<br>d. Eukaryotic cells use a different code to specify the amino acids in proteins than prokaryotic cells.<br>e. All of the above<br>f. A and B<br>g. C and D
	Understand	What physical part of the carbohydrate ends up in ATP during the process of cellular respiration?<br>a. Carbon<br>b. Oxygen<br>c. Electrons<br>d. Phosphate group<br>e. All of the above<br>f. A and B<br>g. A, B, and C<br>h. **None of the above**
	Apply	According to Tree A, is Rolfo more closely related to Jabba or to Midipi?<br>a. Jabba<br>b. **Midipi**<br>c. Rolfo is equally related to Jabb and Midipi<br>d. It's impossible to know without traits labeled on the tree<br>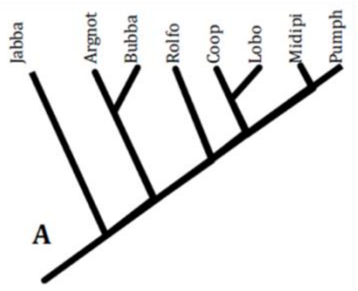
High-order cognitive skills (HOCS)	Apply	If the sequence from the previous question (3' GATGTTACCAAGCATAGTATG 5') were mutated to the following, this would be what type of mutation?<br>3' GATGTTACCA**G**GCATAGTATG 5'<br>a. Missense<br>b. Nonsense<br>c. **Silent**<br>d. Frameshift
	Analyze	You want to know what your blood type is, but you are deathly afraid of needles and don't want to be tested. You ask your parents and grandparents what their blood types are in order to figure it out yourself. Your mom is B+, and her parents are AB- and AB+. Your dad didn't know his blood type, but his parents are both O-. What is your blood type?

		a. AB+<br>b. AB-<br>c. B+<br>d. B-<br>e. O+<br>f. O-<br>g. Could be A or B<br>h. **Could be C or D**<br>i. Could be E or F<br>j. Could be D or F
	Evaluate	Which of the following would be the best control group for an experiment testing whether or not a certain drug prevents cataracts in females over the age of 65?<br>a. male subjects over the age of 65<br>b. **female subjects over 65 that are given placebos**<br>c. female subjects younger than 65<br>d. alternative drugs that are suspected to prevent cataracts
